# Enhanced Degradability of Thiol–Ene Composites through the Inclusion of Isosorbide-Based Polycarbonates

**DOI:** 10.1021/acsami.4c09626

**Published:** 2024-07-20

**Authors:** Jorge San Jacinto Garcia, Natalia Sanz del Olmo, Daniel J. Hutchinson, Michael Malkoch

**Affiliations:** Royal Institute of Technology, School of Chemical Science and Engineering, Department of Fibre and Polymer Technology, KTH, Teknikringen 56-58, 100 44 Stockholm, Sweden

**Keywords:** composites, thiol−ene click chemistry, polycarbonates, degradability, bone fixation

## Abstract

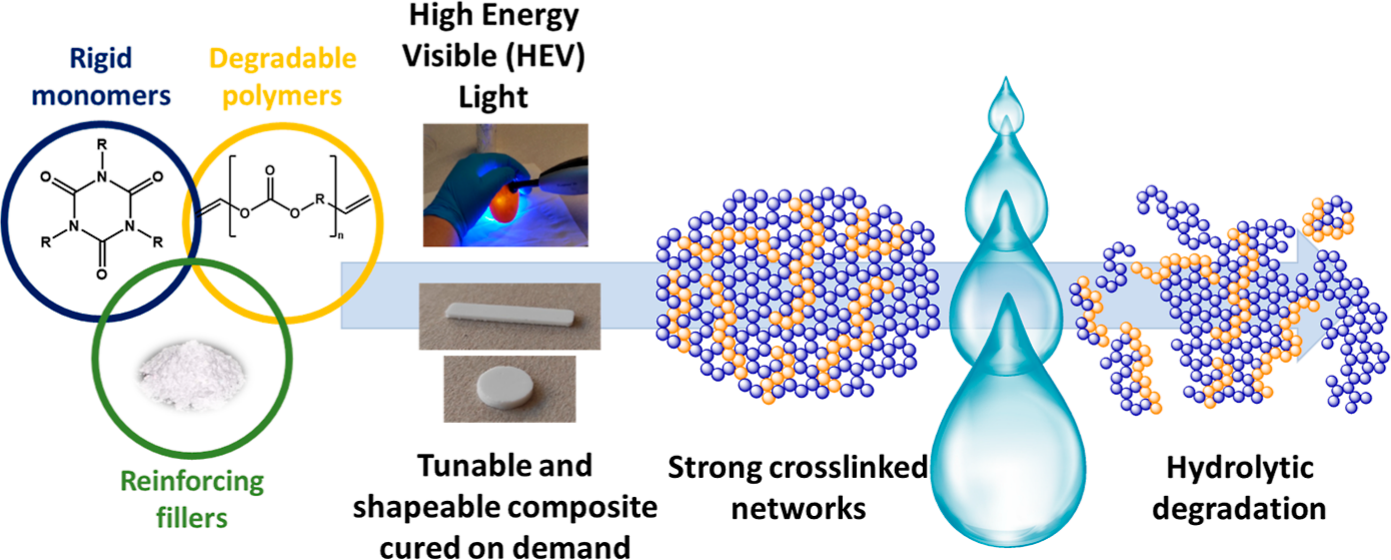

Open reduction internal fixation metal plates and screws remain the established standard-of-care for complex fracture fixation. They, however, have drawbacks such as limited customization, soft-tissue adhesions, and a lack of degradation. Bone cements and composites are being developed as alternative fixation techniques in order to overcome these issues. One such composite is a strong, stiff, and shapeable hydroxyapatite-containing material consisting of 1,3,5-triazine-2,4,6-trione (TATO) monomers, which cures through high energy visible light-induced thiol–ene coupling (TEC) chemistry. Previous human cadaver and in vivo studies have shown that patches of this composite provide sufficient fixation for healing bone fractures; however, the composite lacks degradability. To promote degradation through hydrolysis, new allyl-functionalized isosorbide-based polycarbonates have been added into the composite formulation, and their impact has been evaluated. Three polycarbonates with allyl functionalities, located at the termini (aPC1 and aPC2) or in the backbone (aPC3), were synthesized. Composites containing 1, 3, and 5 wt % of aPCs 1–3 were formulated and evaluated with regard to mechanical properties, water absorption, hydrolytic degradation, and cytotoxicity. Allyl-functionalized polycaprolactone (aPCL) was synthesized and used as a comparison. When integrated into the composite, aPC3 significantly impacted the material’s properties, with the 5 wt % aPC3 formulation showing a significant increase in degradation of 469%, relative to the formulation not containing any aPCs after 8 weeks’ immersion in PBS, along with a modest decrease in modulus of 28% to 4.01 (0.3) GPa. Osteosyntheses combining the aPC3 3 and 5 wt % formulations with screws on synthetic bones with ostectomies matched or outperformed the ones made with the previously studied neat composite with regard to bending stiffness and strength in four-point monotonic bending before and after immersion in PBS. The favorable mechanical properties, increased degradation, and nontoxic characteristics of the materials present aPC3 as a promising additive for the TATO composite formulations. This combination resulted in stiff composites with long-term degradation that are suitable for bone fracture repair.

## 1 Introduction

Bone fractures are a common issue in society, and their incidence is forecast to rise further due to an increasingly aging global population.^1^ Fractures can occur throughout daily life due to falls, accidents, or injuries, leading to a painful experience for the patient and possible disabilities if they are not treated successfully. While simple fractures can be treated conservatively with a splint or an external cast, complex or comminuted fractures usually require surgery and the use of open reduction internal fixation (ORIF) metal plates and screws to maintain reduction and alignment of the bone fragments during healing.^2^ In spite of being the gold standard, the ORIF method has limitations that require urgent attention.^3,4^ The rigid nature of the metal plates limits the extent to which they can be contoured to match the fracture’s geometry and the profile of the bone surface, which necessitates the stockpiling of large inventories of differently shaped implants.^5^ Moreover, soft tissue commonly adheres to the implants, affecting joint mobility and increasing the stiffness of the fracture area. These complications may require the removal of the implant in a second surgical procedure, which increases the recovery time, pain, the possibility of long-term disabilities and the economic cost of fracture fixation.^1,6^ These side effects are particularly common in hand fractures where 64% of phalangeal fractures fixated with metal plates and screws affect the joint mobility of the patient,^7^ and between 39 and 44% of middle phalanx fractures treated with ORIF plates result in joint stiffness that require corrective operations.^4,8,9^ Moreover, metal plates have superior mechanics to human cortical bone, creating excessive stress shielding that affects the bone regeneration over time. In this context, biomaterials with similar mechanical properties to human bones are under investigation with the vision to act as fracture fixators that maintain bone alignment throughout the healing process without causing excessive damage to the fractured bone or surrounding tissue.

The disadvantages with ORIF implants could be solved by creating a material that could be shaped to fit any fracture, providing antiadherent properties to soft tissue while having enough strength to hold the fracture during the healing process. Tetranite is a mineral-organic bone glue that hardens over time, providing osteoconductive and bioactive features while holding the fracture, withstanding tensile and shear strengths of 3 MPa.^10^ IlluminOss is another injectable approach for bone stabilization, which is introduced deflated inside the bone and, then, is distended with a monomer that is hardened with blue light, stabilizing the segment.^11^ Another alternative is to replace the plates or conventional screws with degradable alternatives, such as magnesium alloys that could promote osteogenesis and angiogenesis by themselves.^12^ Biopolymers, such as PLGA, have been widely used to create precast implants as a replacement for metal plates. The possibility to combine these polymers with other components that could accelerate the bone healing makes them attractive alternatives to metal alloys.^13^

Our research group has developed strong and injectable composites for bone fixation based on triazine-trione (TATO) monomers and a high degree of hydroxyapatite (HA). Osteosynthesis is achieved by applying and shaping the composite into bone fixation patches in situ and then curing via high energy visible (HEV) light-induced TEC or thiol–yne chemistry (TYC). Screws are used to anchor the patches to the bone fragments. The mechanical properties of these TATO composites can be tuned through the choice of monomers, with flexural modulus values ranging from 52 MPa to 7 GPa.^14−16^ The stiffest of these composites, based on the TATO alkene and thiol monomers 1,3,5-tiallyl-1,3,5-triazine-2,4,6-trione (TATATO) and 1,3,5-triazine-2,4,6-trione, 1,3,5-tris(mercaptopropyl) (TMTATO), falls within the flexural modulus range of human trabecular bone (3–10 GPa).^17,18^ The fluid nature of the composite resins and their use of on-demand curing allows for drop-casting of screws and plates and even 3D printing, making them suitable for potential prosthetic implants.^19^*In vivo* evaluations in rodents with femur fractures have shown that the TATO composites do not induce postsurgical soft-tissue adhesions,^14,15,20,21^ while biomechanical studies on human hand cadavers and animal bones suggest that composites containing TATATO, TMTATO, and HA can maintain alignment of the bone against bending forces experienced during rehabilitation flexion exercises.^22^

While these TATO composites address the issues with customization and soft-tissue adhesions, they showed no signs of degradation after 12 months in vivo in rodents. An injectable osteosynthesis device that could degrade over a sufficiently long time, such that its degradation does not compromise its mechanical strength during bone healing, is currently an unresolved materials challenge as it would reduce the need for implant removal after healing is complete. One strategy for introducing degradability to the TATO composite is the incorporation of hydrolyzable polymers into the cross-linked network upon cross-linking via TEC chemistry. Recently, allyl-functionalized polyester dendrimers have been used as potential degradable cross-linking additives in TATO composites. Adding up to 5 wt % of these dendrimers into an existing TATO formulation did not jeopardize the strength or modulus of the material, but, unfortunately, it only had a very minor impact on hydrolytic degradation after a time period of 8 weeks.^16^ Easier synthetic alternatives could be used to introduce degradation into composite materials such as linear polymers.

Many biopolymers have been widely used in composites to introduce degradation, such as collagen,^23^ gelatin,^24^ or chitosan,^25,26^ as well as synthetic polymers such as polyvinylpyrrolidone,^27^ polyesters, including polylactic acid^28−30^ and polycaprolactone,^31−33^ and polycarbonates.^34^ Among all these candidates, polycarbonates stand out due to their higher modulus, compared to biopolymers, and faster degradation, compared to polyvinylpyrrolidone. Moreover, the degradation of polycarbonates into carbon dioxide and diols does not cause any localized pH changes, in contrast to polyesters, such as polylactic acid and poly(lactic-glycolic) acid, which release acidic monomers upon degradation that can damage soft tissue.^35,36^ The most currently used polycarbonates are based on bisphenol A (BPA) due to their varied applications.^37^ BPA is commonly used as a strength provider; the bulky and rigid benzene groups present in its structure create stiff and rigid polymers with good mechanical properties.^38^ However, reports have shown that the use of BPA in products coming in contact with humans, such as food packaging, could lead to various health issues due to BPA leach out.^39^ There is an effort to increase the use of aliphatic polycarbonates made from renewable monomers due to their unique combination of biodegradability and biocompatibility that promote their use for biomaterials.^40,41^ A promising alternative to BPA is isosorbide, which is an attractive building block due to its rigidity and nontoxicity, that produces polymers with high *T*_g_ and modulus.^42^ These polymers can be easily produced with green chemistry approaches such as 1,1′ carbonyldiimidazole (CDI) activation, using inorganic catalysts (CsF), which enhances the nucleophilicity of the hydroxyl groups, allowing the use of milder reaction conditions.^43,44^ Cross-linked networks incorporating isosorbide polycarbonates have displayed a slow degradation over time under physiological conditions, with almost no alteration of their structure after 60 days.^45^ This is ideal for bone fixation applications, as the composite’s mechanical integrity must not be compromised before the new remodeled bone (hard callus) is formed, which occurs within 3–5 weeks.^46^

**Scheme 1:**
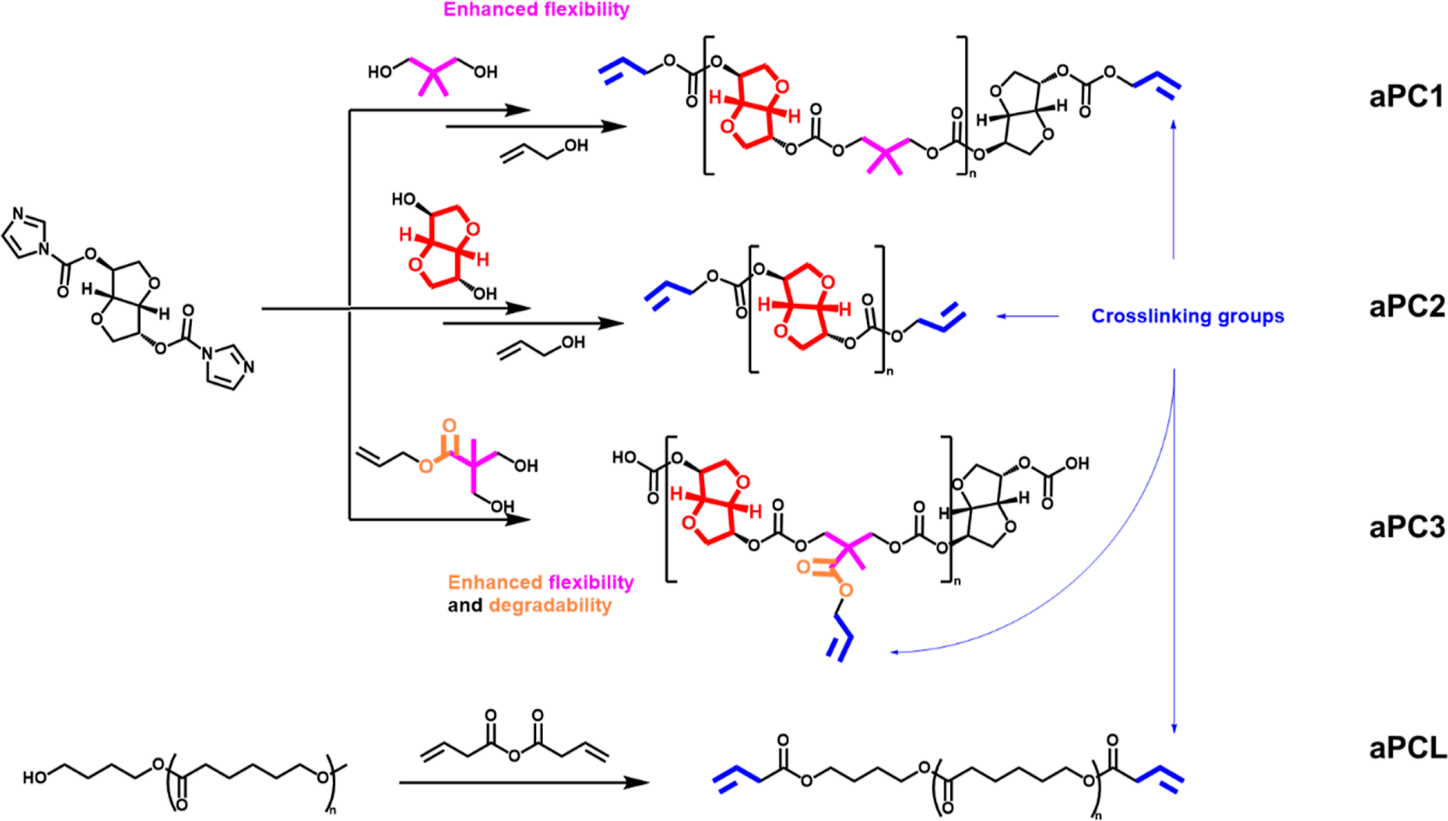
Allyl Polycarbonate Synthetic Strategy Using Step-Growth Polymerization for Aliphatic Polycarbonates via FPC Polymerization^44^ and PCL Functionalization

Herein, we present a new generation of TATO HA composites for use in bone fixation that show enhanced hydrolytic degradation in vitro due to the incorporation of isosorbide-based polycarbonates into their polymeric phase. Three different low-molecular-weight isosorbide-based polycarbonates with allyl functional groups were synthesized via fluoride-promoted carbonylation (FPC) polymerization and esterification (FPE, Scheme 1)^44^ reactions. They were then mixed with composite mixtures containing TATATO, TMTATO, and HA. PCL was also decorated with terminal allyl groups (aPCL) and used as a comparison to the polycarbonates, as was the neat composite, which did not contain any degradable polymer. The influence of the placement of the allyl groups in the polycarbonates and the weight percentage with which they were added to the composite formulations was investigated with respect to mechanical properties, water absorption, in vitro degradation, and cytotoxicity toward human keratinocytes (HaCaT) and mouse monocyte/macrophage-like cells (Raw 264.7).

## 2 Materials and Methods

### 2.1 Materials

The following chemicals were purchased from commercial sources and were used as received unless otherwise noted. Sodium bicarbonate, magnesium sulfate (99%), 2,2 dimethoxypropane (for synthesis), p-toluene sulfonic acid (for synthesis), allyl alcohol (>99%), sodium bisulfate (technical grade), isosorbide (98%), butenoic acid (97%), celite 545, neopentyl glycol (99%), cesium fluoride (99%), phosphate buffered saline (pH = 7.4, tablets), 4-(dimethyl amino) pyridine (DMPA) (98%), 1,3,5-tryallyl-1,3,5-triazine-2,4,6(1*H*,3*H*,5*H*)-trione (TATATO) (98%), hydroxyapatite (reagent grade), and lithium phenyl(2,4,6-trimethylbenzoyl) phosphinate (TPO) (97%) were purchased from Sigma-Aldrich Sweden AB. Thioacetic acid (98%) and Dowex 50WX2 50–100 were purchased from Thermo Scientific. Hydrochloric acid (37%), methanol (HPLC-gradient reagent), acetone, and pyridine (99.9%) were purchased from VWR Chemicals. Ethyl acetate, heptane, dichloromethane (for analysis), diethyl ether (for analysis), and *N*,*N*′-dimethylformamide (for gas chromatography) were purchased from Merck. PCL 4k and 2,2-bis(methylol)propionic acid (bis-MPA) were purchased from Perstorp. *N*,*N′*-Dicyclohexylcarbodiimide (DCC) (99%) was purchased from Acros Organics. 1,1-Carbonylimidazole (CDI) (97%) was purchased from TCI Chemicals. 1,3,5-Triazine-2,4,6-trione 1,3,5-tris(mercaptopropyl) (TMTATO) was synthesized according to literature procedures.^48^

### 2.2 Instrumentation

#### 2.2.1 Size Exclusion Chromatography

A TOSOH EcoSECHLC-8320GPC system equipped with an EcoSES RI detector and three columns from PSS GmbH was used (PSS PFG 5 μm; Microguard, 100 and 300 Å). The mobile phase was DMF with 0.01 M LiBr (0.2 mL min^–1^) at 50 °C using a conventional calibration method with narrow linear poly(methyl methacrylate) (PMMA) standards.

#### 2.2.2 Nuclear Magnetic Resonance

A Bruker 400 MHz nuclear magnetic resonance (NMR) instrument was used to perform the experiments. ^1^H NMR and ^13^C NMR spectra were recorded at 400 and 101 MHz, respectively. Samples were analyzed in CDCl_3_, and the chemical shift values were referenced to the residual solvent peak at 7.26 ppm for ^1^H NMR and 77.0 ppm (middle peak) for ^13^C NMR spectra. All spectra were analyzed with MestreNova software (v 14.2.0-26256, Mestrelab Research S.L.).

#### 2.2.3 Differential Scanning Calorimetry

A Mettler Toledo DSC820 instrument with a heating and cooling rate of 10 °C min^–1^ was used. The data were collected by starting from 20 °C and heating to 200 and 250 °C for copolycarbonate and homopolycarbonate, respectively. Analyses regarding midpoint *T*_g_ were performed on the second heating scan.

#### 2.2.4 Fourier Transform-Raman

A portable i-Raman Plus spectrometer (model: BWS465–785S, B&W TEK) was used to determine the complete conversion of thiols and alkenes upon curing. The measurements were applied to the neat composite and formulations with the highest wt % of degradable polymers. A total of 48 scans (laser wavelength: 785 nm, laser power: 340 mW, integration time: 1000 ms) were used per spectrum. The raw data were analyzed on BWSpec software and plotted in Origin 2020 (Academy). The carbonyl shift at 1760 cm^–1^ was used to normalize the spectra. Thiol (2575 cm^–1^) and alkene (1645 cm^–1^) shifts were compared to confirm the full monomer conversion upon HEV-induced TEC.

#### 2.2.5 Scanning Electron Microscopy

Scanning electron microscopy (SEM) was conducted with a low-vacuum Hitachi Tabletop SEM TM-1000 (Japan), equipped with a BSE detector and a W filament. The voltage used was 15 kV, and pictures were taken with a magnification of 50× and 600×. Samples were coated with a Cressington 208HR sputter coater with a Pd target, using coating thicknesses between 3 and 5 nm to increase the conductivity of the samples.

#### 2.2.6 Dynamic Mechanical Analysis

The glass transition temperatures (*T*_g_) and onset points of the composites were measured by a Dynamic Mechanical Analyzer (Q800, T.A. Instruments, USA) in thin film/tensile mode. The materials had approximate dimensions of 12 mm in length, 6 mm in width, and 1.5 mm in thickness. The samples were tested under either dry or wet conditions. A temperature ramp method with a heating rate of 3 °C/min was used with a temperature span between 10 and 130 °C. A strain of 0.1% was induced with a frequency of 1 Hz.

#### 2.2.7 Contact Angle

A contact angle meter (Theta Lite, Biolin Scientific) was used to determine the hydrophobicity and hydrophilicity of the different formulations. A drop of water (4 μL) was deposited on the surface of a composite disk, and the angle of the water drop was measured. Three samples were tested for each formulation and time point.

### 2.3 Methods

#### 2.3.1 Formulation of Composite Materials

The composites were prepared as described previously in the literature.^15^ aPCs 1–3 and aPCL were mixed together in a glass vial with Cat57, TMTATO, and TATATO. Stirring and gentle heating were applied until a homogeneous solution was obtained. After cooling, the vial was protected from light, and TPO and HA were added. The mixture was stirred until a homogeneous, white, viscous fluid was obtained.

#### 2.3.2 Curing of Composite Materials

The resins were cured by a portable high-performance curing LED lamp (Bluephase 20i, Ivoclar Vivadent AG, Leichtenstein) with spectral wavelengths of 385–515 nm (dominant wavelengths of 400 and 470 nm) and a light intensity of 2000 mW/cm^2^. At least two pulses (5 s/pulse) of LED treatment were applied per cm^2^ on both sides of the composite surface to ensure full conversion of monomers.

#### 2.3.3 Composite Porosity

The porosity of the cross section of the beams was determined by using ImageJ (NIH) software analysis of the SEM pictures of the cross-sectional area of the beams. To determine the % of porosity, the total area of the pores was determined, excluding the pores with a lower size than 3 × 10^–4^ mm^2^. This area was then divided by the cross-sectional area or the beam and multiplied by 100.

#### 2.3.4 Three-Point Bending Testing

Mechanical analysis was conducted on rectangular samples of dimensions 35 × 6 × 1.5 mm (length × width × thickness) after curing (dry) and after being immersed in 20 mL of phosphate buffered saline solution (PBS; pH = 7.4) at 37 °C for 2, 4, 6, or 8 weeks (wet). All samples were allowed to acclimate to the testing temperature of 20 °C before testing began. The wet samples were then taken out from their solutions, and excess water on their surface was removed with tissue paper. Both dry and wet samples were tested on an Instron 5566 double column universal testing machine (Instron Korea LLC) with a load cell of 500 N and a crosshead speed of 1 mm/min, a preload of 0.1 N, and a preload speed of 0.5 mm/min. The center-to-center distance of the lower contacts was set to 30 mm, and all the measurements were conducted at 20 °C with a relative humidity of 50%. The data were analyzed and collected by Bluehill software. The flexural modulus was calculated by eq 1, where *L* is the lower contacts’ distance, *m* is the slope at the initial elastic region of the load and displacement curve, *w* is the width of the beam, and *d* is the thickness of the beam. Five samples were tested for each formulation and time point.
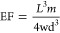
1

#### 2.3.5 Four-Point Bending Testing

Mechanical analysis was conducted on synthetic bone substrates (cylindrical rods of PEEK with a diameter of 10 mm) with ostectomies of 10 mm in length that were fixated with composite patches containing neat, aPC3 3 or 5 wt %. The patches were anchored to the substrate with four bicortical screws (1.5 mm diameter) using a previously reported procedure known as AdhFix.^15^ The osteosyntheses were evaluated with four-point bending under dry conditions or after being soaked in PBS (pH = 7.4) at 37 °C for 2 weeks (wet conditions). All samples were allowed to acclimate to the testing temperature of 20 °C before testing. The wet samples were then taken out from their solutions and excess water on their surface was removed with tissue paper. Both dry and wet samples were tested on an Instron 5566 double column universal testing machine (Instron Korea LLC) with a load cell of 500 N, a crosshead speed of 1 mm/min, a preload of 0.5 N, and a preload speed of 0.5 mm/min. The support span was set to 45 mm, and the loading span was set to 15 mm. All of the measurements were conducted at 20 °C, with a relative humidity of 50%. The data were analyzed and collected by Bluehill software. Five samples were tested for each formulation and time point. Bending stiffness (K) was calculated at the initial elastic region of the load vs displacement curve, using eq 2, where F is the force and d is the displacement. The bending structural stiffness (EIe) was calculated with eq 3, where h is the loading span distance, a is the center span distance, and K is the bending stiffness. Bending Strength (BS) was calculated using eq 4, where P is the proof load and h is the loading span distance. The BS normalized with the cross-sectional area (CSA) of the fixation patch was determined using eq 5, where BS is the bending strength, w is the width, and t is the thickness of the fixation patch.
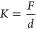
2
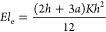
3
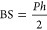
4
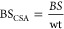
5

#### 2.3.6 Water Absorption and Degradation Measurements

Water absorption and degradation tests were conducted on the neat composite as well as all concentrations (1, 3, and 5 wt %) of aPCs and aPCL. Five samples were made in a plastic mold of diameter 12.5 mm and thickness 1.5 mm for each concentration, formulation, and time point (2, 4, 6, and 8 weeks). Thereafter, samples were placed in a desiccator in an oven at 37 °C until the dried weight was constant within 0.1 mg (*m*_1_). Afterward, they were stored in separated vials filled with enough PBS solution (pH 7.4) to be completely submerged, at 37 °C. For water absorption, the mass of the samples was monitored every 2 weeks during an 8 week period. The samples were taken out from the oven and washed with deionized water, and the excess water was removed with tissue paper. The mass was then recorded (*m*_2_) and the samples were returned to the oven until the last time point was reached. For degradation, at time points of either 2, 4, 6, or 8 weeks, the disks were taken out from the PBS buffer and completely dried at 140 °C until they obtained a constant dried weight (*m*_3_). Water absorption and degradation were calculated using eq 7
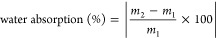
6
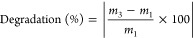
7

#### 2.3.7 Cytotoxicity Assays

A monolayer of human epidermal keratinocytes (HaCaT) and mouse monocyte/macrophage-like cells (Raw 264.7) was used for the cytotoxicity tests. These cell lines were maintained in tissue culture flasks at 37 °C in 5% CO_2_ with Dulbecco’s Modified Eagle Medium (DMEM), supplemented with 10% (v/v) Fetal Bovine Serum, 100 IU mL^–1^ penicillin, and 100 μg mL^–1^ streptomycin. A solution of 100 μL of the cells at a concentration of 5 × 10^5^ cells mL^–1^ was harvested in 96-well plates for the cytotoxicity test and incubated for 24 h at 37 °C and 5% CO_2_. Meantime, disks made of neat composite and 5 wt % aPCs 1–3 and aPCL containing composites were exposed to EtOH at 70% for sterilization for 1 h and washed three times with PBS to complete the process with the exposure of those materials to UV light for 15 min. Afterward, the solid material (disks of 12.5 mm of diameter and 1.5 mm of thickness) was transferred into 2 mL of complete DMEM and incubated at 37 °C for 24 h, 1 week, and 2 weeks to get the leach-out medium (the testing medium). Afterward, the old cell culture medium was replaced by 100 μL of the testing medium per well and incubated for 24 h. For each sample medium, six parallel wells were used, and three discs were used for each material’s formulation. Cells without treatment were used as a control, and extracts without cells were used as blank of this experiment. Then, 10 μL of AlamarBlue agent was applied and incubated for 4 h at 37 °C in 5% CO_2_. Finally, the fluorescent intensity was measured with a plate reader (Tecan Infinite M200 Pro) at the wavelength of 560/590 nm (excitation/emission). Formulations showing less than 70% of cell viability were considered as toxic following the ISO10993-5:200947 standard.^47^

## 3 Results and Discussion

To introduce degradability to the current TATATO, TMTATO, and HA composite, we sought out the assessment of linear aPCs as attractive cocomponents. We hypothesized that the presence of numerous hydrolyzable carbonate bonds in aPC structures combined with the decoration of these polymers with allyl functionalities would enhance degradation of the composite as the hydrolyzable aPCs would be covalently integrated into the otherwise nonhydrolyzable polymeric matrix. A low molecular weight was desired for the aPCs in order to facilitate their integration into the composite mixture and retain the processability of the final composite resin. The entanglement of the linear polymeric chains could reinforce the material, decreasing its brittleness and making it more resistant to micro and macro motions. Isosorbide-based polycarbonates have shown *T*_g_ values well above 100 °C, so their incorporation into the existing TMTATO, TATATO, HA system was not expected to significantly reduce its mechanical properties under physiological conditions, which is an important consideration given the intended application of internal bone fixation.^43^ PCL, a common biopolymer known for its biodegradability, was also decorated with allyl functionalities as an ester-based compound comparison.

### 3.1 Synthesis of Polycarbonates as Degradable Additives for the Formulation of Composites

Different allyl-functionalized homo- and copolycarbonates were synthesized via step-growth polymerization between bis-carbonylimidazolide-activated isosorbide and different diols (Scheme 1). aPC1 contained both isosorbide and neopentyl glycol in its structure, for enhanced flexibility to promote entanglement of the polycarbonate within the composite matrix. aPC2 consisted of repeating isosorbide units, resulting in a more rigid polymer. aPC3 was a copolymer of isosorbide and allyl-functionalized 2,2-bis(methylol)propionic acid (bis-MPA), resulting in additional hydrolyzable ester groups throughout its polymer backbone to improve water absorption and degradability. All three polycarbonates contained allyl groups for cross-linking with the TATO-based alkene and thiol monomers via HEV-induced TEC chemistry. However, aPCs 1 and 2’s allyl groups were located at the termini of the polymers while aPC3 contained pendant allyl groups along its backbone for better integration into the polymeric network. Additionally, conventional 4k-PCL was functionalized with allyls through a reaction with 3-butenoic anhydride. All of these polymerization reactions were successfully achieved by using FPC chemistry with CsF as an inorganic polymerization catalyst. Different solvents (DCM, CHCl_3_, DMF, and THF) and temperatures (rt and 60 °C) were experimented with during the synthesis of PCs 1 and 2 (Table 1). DCM and rt were chosen as the preferred conditions for synthesis of PC1, PC2, and aPC3 as they resulted in consistently low-molecular-weight polymers, which were desired in order to facilitate the incorporation of the polymers within the composite formulation.

**Table 1:** Molecular Weight, Polydispersity, *T*_g_, and Yield of the Polymers Made through FPC of Bis-Carbonyldiimidazolide Isosorbide and Neopentyl or Isosorbide Using Different Solvents^a^

monomer A	monomer B	solvent	temperature (°C)	*M*_n_ (kDa)	*D*	*T*_g_ (°C)	yield (%)
isosorbide	neopentyl	DCM	r.t	3.41 (0.03)	2.45 (0.03)	88 (3)	41
isosorbide	neopentyl	CHCl_3_	r.t	9.41 (0.59)	1.96 (0.05)	111 (1)	48
isosorbide	neopentyl	DMF	60	6.01 (0.08)	1.73 (0.02)	98 (0)	70
isosorbide	neopentyl	THF	60	5.12 (0.45)	1.60 (0.00)	97 (1)	45
isosorbide	isosorbide	DCM	r.t	5.27 (1.53)	1.97 (0.37)	129 (9)	72
isosorbide	isosorbide	CHCl_3_	r.t	9.72 (0.73)	1.61 (0.07)	139 (9)	73
isosorbide	isosorbide	DMF	60	13.50 (0.12)	1.42 (0.02)	165 (2)	69
isosorbide	isosorbide	THF	60	25.83 (3.67)	1.49 (0.04)	130 (6)	70

a Data presented as mean with standard error of mean shown in parentheses. *n* = 3 for each polymer.

**Figure 1:**
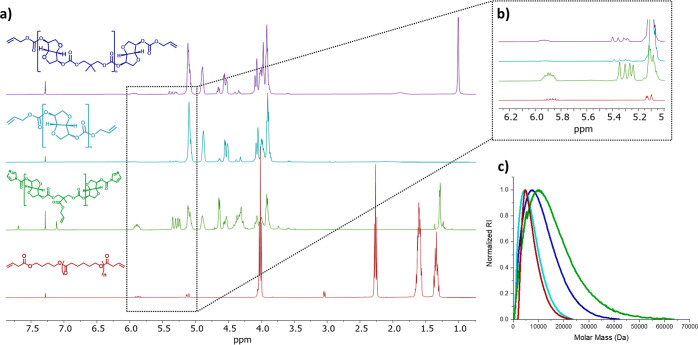
(a) Stacked ^1^H NMR spectra of aPC1 (blue), aPC2 (turquoise), aPC3 (green), and aPCL (red). (b) Stacked ^1^H NMR magnification of the peaks due to the allyl functionalities. (c) SEC chromatograms of aPCs and aPCL.

The reactions were monitored by ^1^H and ^13^C NMR spectroscopy. The FPC step was determined to be complete upon the disappearance of the imidazolide carbon peak at 148.1 ppm and the formation of new carbon peaks from the carbonate bonds at approximately 153.7 ppm for all PCs. The postfunctionalization reactions of PCs 1 and 2 with allyl alcohol were monitored by ^1^H NMR spectroscopy (Figure 1a,b).

The consumption of the imidazolide peaks at 8.12, 7.40, and 7.07 ppm and the appearance of allyl functionality peaks at 5.90 and 5.32 ppm confirmed the success of the reaction. For PCL functionalization, the complete reaction was confirmed by ^1^H NMR spectroscopy with the shift of the peak corresponding to the –CH_2_–OH proton from 3.62 to 3.02 ppm. The appearance of new allyl peaks at 5.85 and 5.10 ppm verified PCL functionalization. SEC showed that aPCs 1–3 were obtained at low molecular weights of 3.79, 2.91, and 4.21 kDa, respectively, while the molecular weight of aPCL was 4.89 kDa (Figure 1c). The polydispersity ranged from 1.2 for aPCL to 2.2 for aPC3. Differential scanning calorimetry showed that the glass transition temperatures (*T*_g_) of aPCs 1–3 were 112, 152, and 91 °C, respectively, all well above the physiological temperature. The T_g_ of aPCL was significantly lower at 52 °C (Table S1).

### 3.2 Formulation of Biodegradable Composites

aPCs 1–3 and aPCL were added at different weight percentages (1, 3, and 5 wt %) to the established TATO, TMTATO, and HA fracture fixation composite formulation (Table S2). As these aPCs were powders, their introduction into the composite mixture required a melting process that, after cooling, increased the viscosity of the final system. Due to this increment in viscosity, 5 wt % was found to be the maximum concentration at which these polymers could be included while maintaining the processability of the composite resins. As a mean to assess the impact of these polymer additives on the overall performance of the composites, three formulations were sought out for each system, i.e., 1 wt % as the lowest and 3 and 5 wt % as the highest amount.

**Figure 2:**
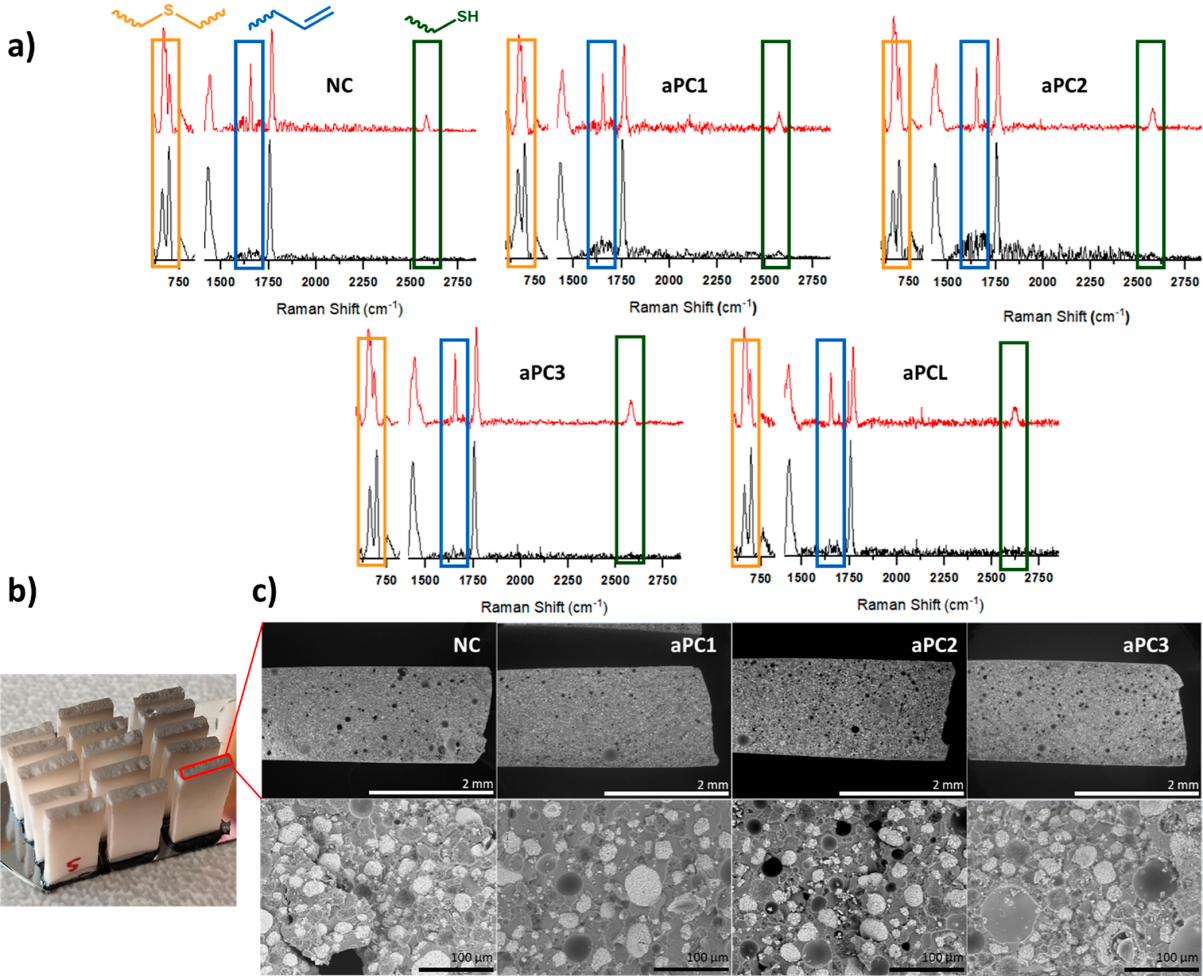
(a) FT-Raman spectra of uncured (red) and cured (black) neat composite and 5 wt % formulations of aPCs and aPCL. Main signals: thioether (orange), alkene (blue), and thiol (green). (b) SEM samples, already coated. (c) Cross-sectional pictures of the neat composite and aPCs 1–3 5 wt % formulations (50×) and magnifications (600×).

The presence of aPCs and aPCL, even at the highest concentration of 5 wt %, did not affect the efficiency of the TEC curing reaction, as FT-Raman spectroscopy showed the complete consumption of the alkene and thiol peaks at 1630–1660 and 2560–2600 cm^–1^, respectively, upon HEV irradiation (Figure 2a). Curing also resulted in the increase of the intensity of the peak at 695 cm^–1^, corresponding to thioether stretching. Additionally, aPCs’ incorporation did not affect the compatibilization of the polymeric matrix with the inorganic filler (Figure 2c).

### 3.3 Mechanical Performance in Dry Conditions

**Figure 3:**
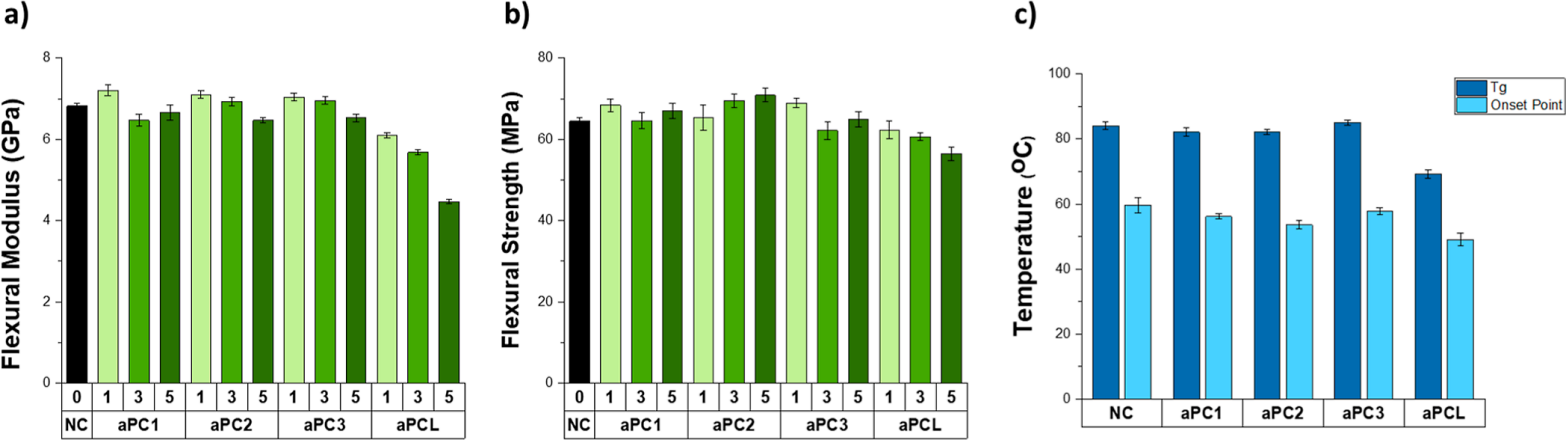
(a) Flexural modulus and (b) flexural strength of the neat composite and 1, 3, and 5 wt % aPCs 1–3 and aPCL containing composites in dry conditions. (c) *T*_g_ and onset point of the neat composite and 5 wt % aPC1–3 and aPCL containing composites in dry conditions. Error bars represent standard error of the mean (*n* = 5).

The addition of 1 wt % of aPCs 1–3 to the composite increased its flexural modulus (*E*_f_) from 6.8 (0.1) to 7.2 (0.1), 7.1 (0.1) and 7.0 (0.1) GPa, respectively (Figure 3). Increasing the concentration of aPCs 1–3 to 3 or 5 wt % either had no effect on the modulus or caused it to slightly decrease in the case of aPCs 2 and 3. This phenomenon might have been related to the increase in the viscosity of the final formulation upon increasing the aPC concentration, which could have resulted in an increased occurrence of imperfections, such as air bubbles, during the fabrication and curing of the samples. Another parameter that has an apparent impact on the mechanics is the replacement of TATATO, as a component, with the more flexible polymers, which result in lower cross-linking density of the final composites. The addition of the aPCs to the composite did not have a significant impact on the flexural strength, with all composites having strength values between 55 and 70 MPa. The inclusion of 5 wt % of aPCs 1–3 did not negatively affect the onset point or *T*_g_ of the composites, with all materials having onset points above 50 °C. This was a positive result considering the intended use for the composites requires them to maintain their rigidity at the physiological temperature. In contrast, the concentration of aPCL was clearly correlated with a significant decrease in modulus, and its inclusion at 5 wt % caused the T_g_ of the composite to decrease by more than 10 °C; however, as with aPCs 1–3, aPCL did not affect the strength of the composite.

### 3.4 Water Absorption and Degradation Evaluation

**Figure 4:**
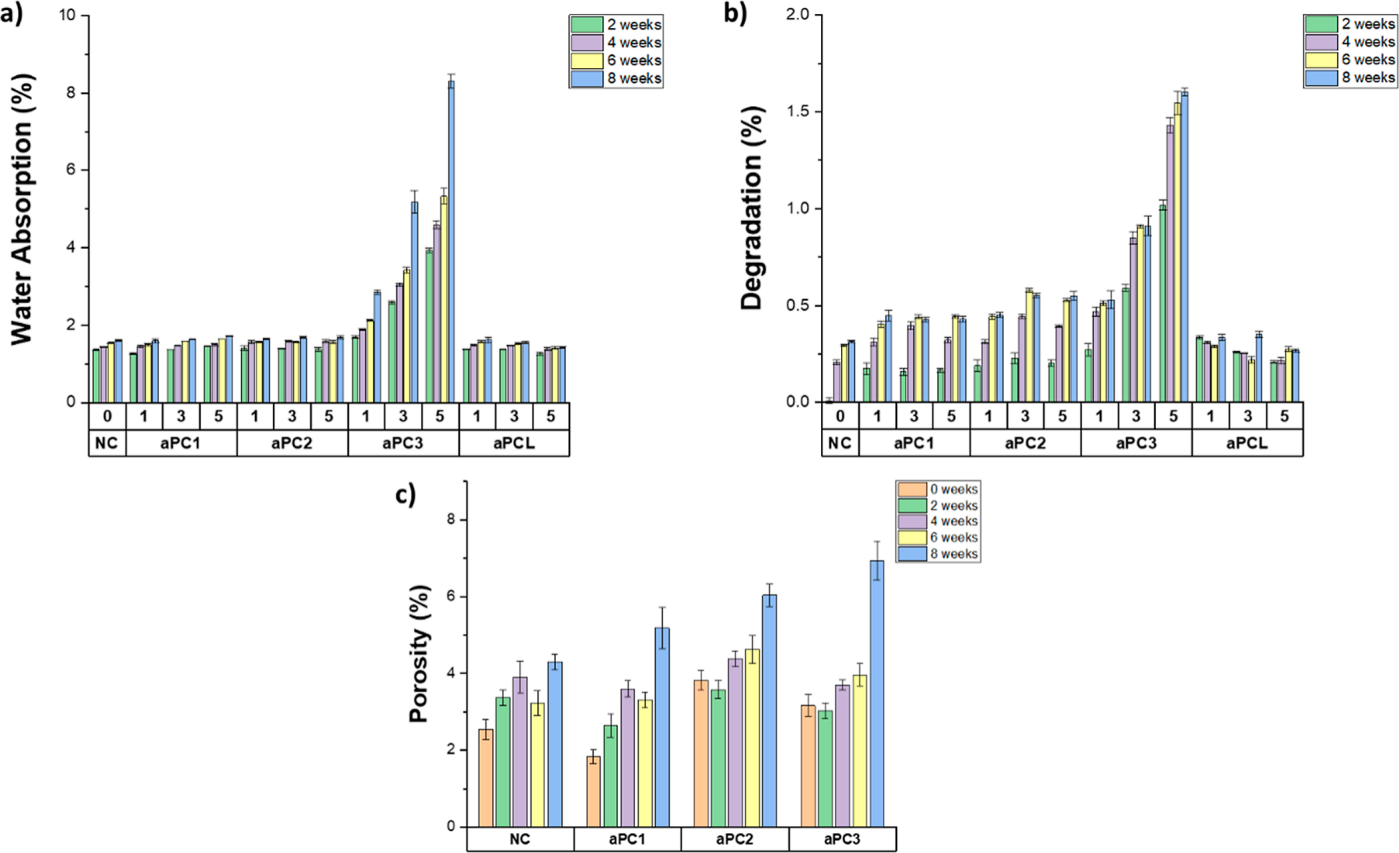
(a) Water absorption and (b) degradation of the neat composite and 1, 3, and 5 wt % aPCs 1–3- and aPCL-containing composites over a period of 8 weeks. (c) Porosity of the neat composite and 5 wt % aPCs 1–3-containing composites. Error bars represent standard error of the mean (*n* = 5).

To evaluate the water absorption and mass loss over time due to hydrolysis, disks of the neat composite and composites containing aPCs 1–3 and aPCL at different weight percentages were soaked in PBS buffer (pH = 7.4) at 37 °C under static conditions and weighed after 2, 4, 6, and 8 weeks (Figure 4a). The behavior of the composites with regard to water absorption did not appear to be impacted by the addition of aPCs 1 or 2. However, when aPC3 was added to the composite formulation, the water absorption increased significantly in a manner that was both time-dependent and impacted by the aPC3 concentration. The addition of 5 wt % aPC3 into the composite increased the water absorption after 2 weeks by a factor of 3, from 1.36 (0.01) % for the neat composite to 3.94 (0.06) %. This behavior became more evident when increasing the soaking time: after 8 weeks, the water absorption increased by a factor of 5 from 1.61 (0.02)% for the neat composite to 8.31 (0.18)% for aPC3 5 wt %. The composites containing aPCL showed significantly lower water absorption than those with aPCs 1–3, probably due to the polyester backbone being more hydrophobic than the polycarbonates (Table S3).

These results were in concordance with contact angle measurements, where the prototype and formulations containing 5 wt % degradable polymer were evaluated (Table S3). The composites containing aPCs 1–3 showed similar contact angles of 51 (2), 59 (2), and 50 (2) °, respectively. These values were similar to that of the neat composite, which was 59 (3) °. The aPCL 5 wt % composite was significantly more hydrophobic, with an angle of 73 (1) °, which was expected due to the hydrophobicity of PCL.^48,49^ The hydrophilic nature of all of the composites was likely due to the high HA content. The contact angles were not significantly affected by immersion in PBS, with the values after 8 weeks being either constant or slightly lower than at time 0.

The choice of polycarbonate had a significant impact on the extent to which the composites lost mass from hydrolytic degradation (Figure 4b). After 8 weeks in PBS at 37 °C, the composites containing aPCs 1 and 2 showed a modest increase in degradation relative to that of the neat composite. For aPC1, the degree of degradation was not dependent on the polycarbonate concentration; however, for aPC2, the 3 and 5 wt % formulations showed higher degradation than the 1 wt % composite. The extent of degradation was significantly higher for the formulations containing aPC3: after 8 weeks the mass losses of the 1, 3, and 5 wt % aPC3 formulations were 169, 289 and 469% higher, respectively, than the neat composite formulation. A mass loss of 1.60 (0.02) % was achieved by the 5 wt % aPC3 formulation, compared to just 0.31 (0.01) % for the neat composite. In contrast, the composites containing aPCL showed degradation comparable to or less than that of the neat composite, with the 5 wt % aPCL composite losing only 0.27 (0.01) % of its mass after 8 weeks.

These results were also supported by porosity measurements performed in the cross section of the rectangular composite samples after mechanical testing (Figure 4c). The initial porosity was mostly due to air bubbles trapped in the composite during the curing process. However, the increase in porosity over time as the samples were immersed in PBS was reflective of the degradation of the materials. The incorporation of degradable polycarbonates into the composite increased the porosity of the network over time compared to that of the prototype. Following the same trend seen in degradation results, the neat composite showed the lowest porosity with 4.30 (0.20) % after 8 weeks, followed by aPC1, aPC2, and aPC3, with values of 5.18 (0.54), 6.03 (0.30), and 6.94 (0.51) %, respectively.

### 3.5 Mechanical Performance in Wet Conditions

**Figure 5:**
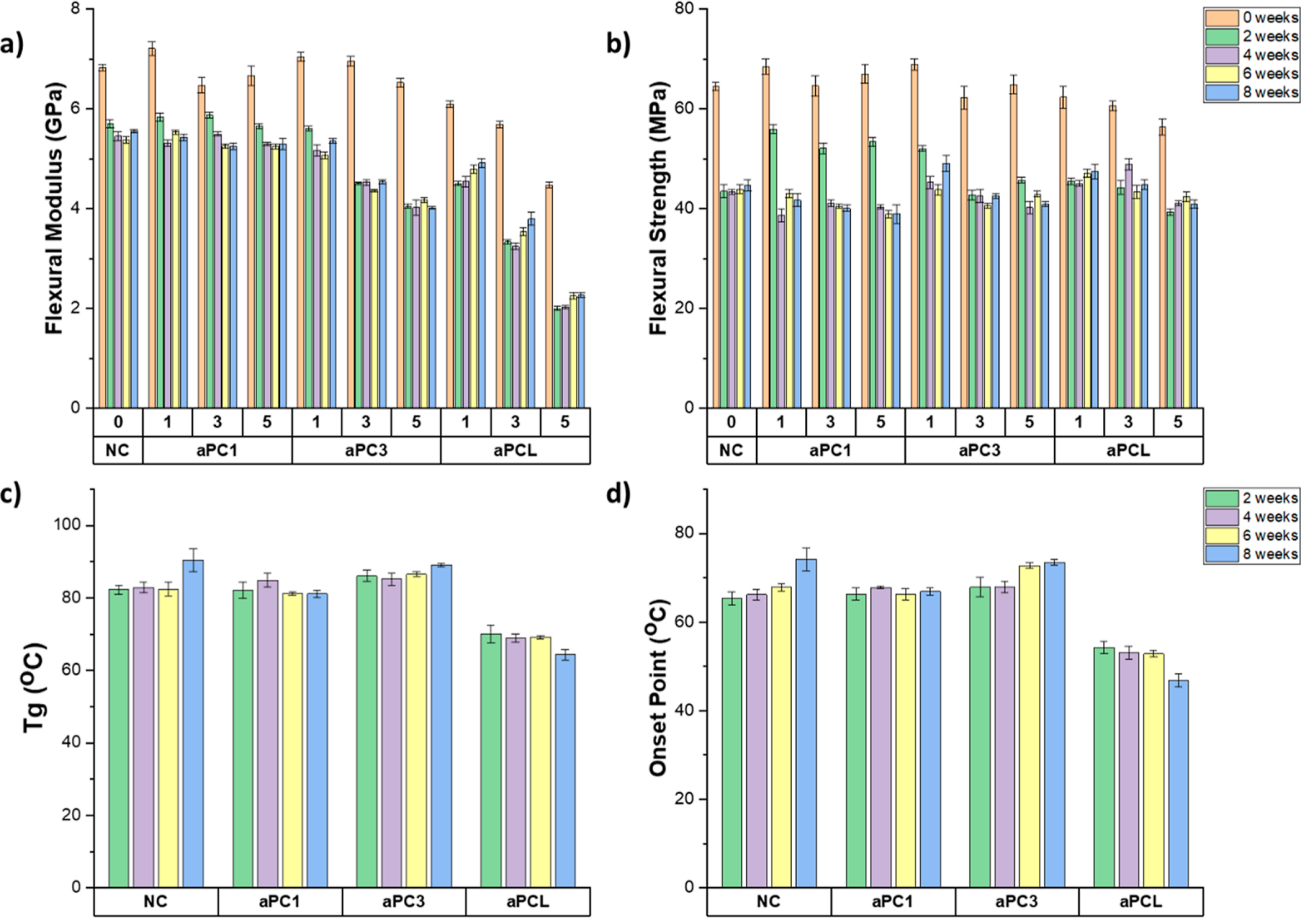
(a) Flexural modulus and (b) flexural strength of the neat composite, aPC1, aPC3, and aPCL under dry and wet conditions. (c) *T*_g_ and (d) onset point of the neat composite, aPC1, aPC3, and aPCL under wet conditions. Error bars represent standard error of the mean (*n* = 5).

Since the intended use of the composite is in a physiological environment, it was important to assess the impact that immersion in PBS at 37 °C had on the mechanical properties of the composites. In general, the flexural modulus of the composites decreased by a modest 1.0–1.4 GPa within 2 weeks, before remaining mostly constant from 2 to 8 weeks (Figure 5a). The decrease in modulus was not affected by the concentration of polycarbonate, with the exception of aPC3, which resulted in increasingly lower modulus results with an increasing aPC3 concentration. This drop in mechanical properties for aPC3 formulations is due to the presence of linear polymers with hydrolyzable carbonate as well as ester groups which, in tandem, can form hydrogen bonding with water, increasing the water absorption. Subsequently, the water acts as a molecular softener, which leads to a decreased flexural modulus. The introduction of flexible PCL resulted in a severe drop in flexural modulus of the aPCL composites when compared with the aPCs 1–3 composites, with decreases of 1.5, 2.3, and 2.5 GPa for the 1, 3, and 5 wt % aPCL composites, respectively. Flexural strength was also affected by water absorption. The neat composite and all aPCL formulations showed a decrease of 21 and 15–16 MPa, respectively, after only 2 weeks in PBS, before stabilizing from 2 to 8 weeks (Figure 5b). However, the aPCs’ formulations showed a slower decrease in strength over 4 weeks in PBS, maintaining better mechanical performance for a longer time, which is crucial in the first weeks of the healing process. The decreases in mechanical properties within the first 2 weeks in PBS are in concordance with the water absorption, the majority of which occurs within 0–2 weeks. Interestingly, the aPC3 formulations maintained their modulus and strength values from 4 to 8 weeks, despite their water absorption and degradation continuously increasing throughout the 8 week period. This means that the degradation between 4 and 8 weeks was not enough to affect the mechanics of the different formulations, not even with aPC3 formulations that exhibited the highest water absorption and degradation. The maintained mechanical properties over this period of time is important as is between weeks 3 and 5 when the bone is remodeled and the hard callus is formed.^46^

**Figure 6:**
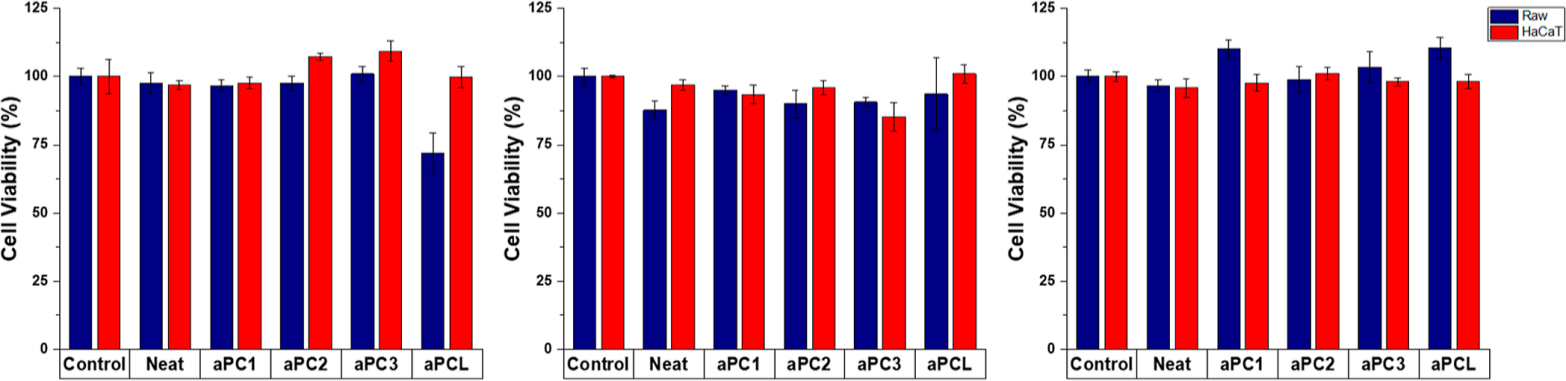
Cell viability of the neat composite and aPCs 1–3 5 wt % and aPCL 5 wt % after 1 day (left), 7 days (middle) ,and 14 days (right) against Raw and HaCaT cells.

*T*_g_ and onset point were not affected by water absorption over time for any of the formulations (Figure 5c,d). The onset points of the aPC containing composites were all above 60 °C even after 8 weeks of being immersed in PBS at 37 °C, so these materials would not be expected to soften under physiological conditions. All formulations containing aPCs showed excellent cytocompatibility with more than 85% of cell viability (Figure 6). Interestingly, only the aPCL formulation after 1 day of incubation showed a cell viability that could be considered as potentially toxic, 72 (7)%. The high cell viability values suggested that the degradation products from the aPCs were nontoxic and indicated that the aPCs’ formulations might be suitable for biomedical applications.

### 3.6 Mechanical Performance as Fracture Fixators

**Figure 7:**
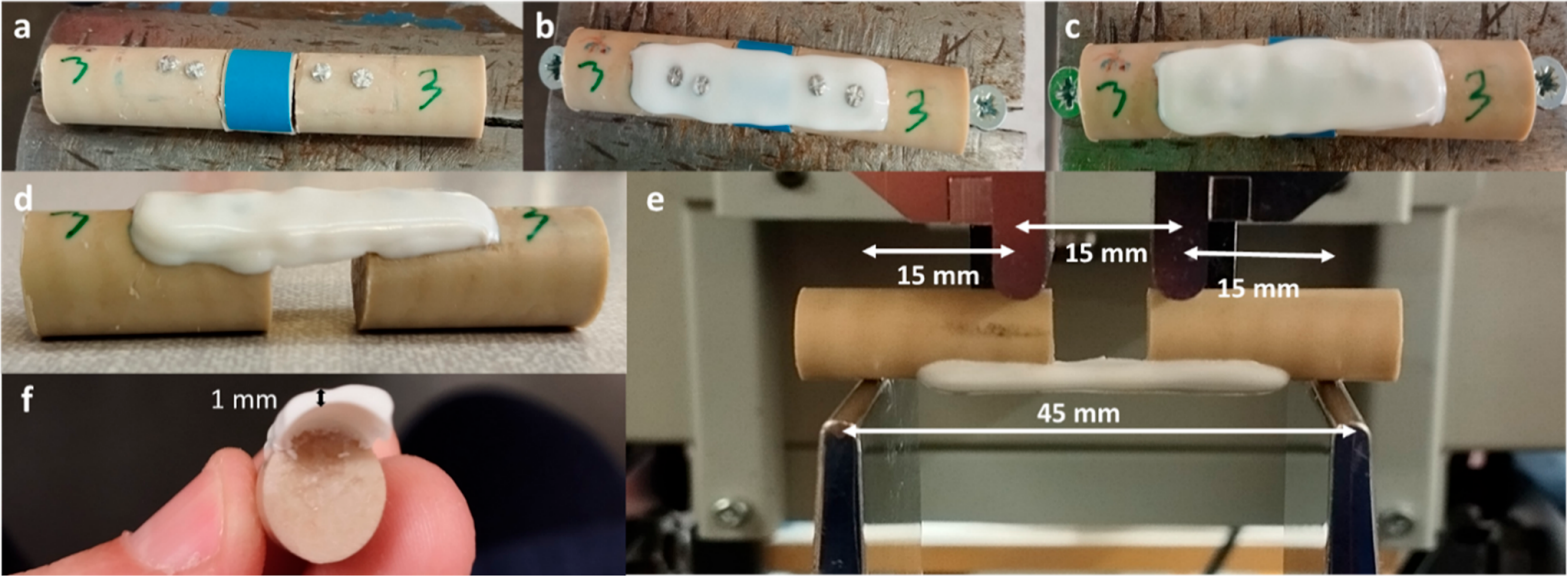
Osteosyntheses of synthetic bone substrates with 10 mm ostectomies were made using the neat and 3 and 5 wt % aPC3 composites. (a) Aligned synthetic bone. (b) First layer of the composite (not covering screws). (c) Second layer of the composite (covering screws). (d) Final osteosyntheses. (e) 4-Point bending test setup. (f) Cross-sectional patch after four-point bending testing.

The results showed that the inclusion of polycarbonates, especially aPC3, into the TATO-based composite formulation increased the hydrolytic degradation of the composite without jeopardizing its stiffness or strength. This suggested that these polycarbonate-infused materials could have potential as strong, degradable bone fixation materials. To further explore their use in this field, the most promising formulations were evaluated as fracture fixators on synthetic PEEK bone substrates with transverse ostectomies (10 mm gap), representing catastrophic comminuted fractures. Osteosynthesis was achieved using the previously described AdhFix method (Figure 7).^15^ Patches of the 3 and 5 wt % aPC3-containing composite were anchored to the bone fractures via screws and subjected to monotonic four-point bending under dry conditions. Moreover, patches of 3 and 5 wt % were tested after 2 weeks soaked in PBS (pH = 7.4) at 37 °C. Patches made with the neat composite were used as a comparison.

All of the osteosyntheses were loaded until failure, which occurred due to fracturing of the composite patch above the ostectomy, Table 2. The bending stiffness of the osteosyntheses containing the aPC3 composites increased from 48.0 (5.3) N/mm for the neat composite to 63.6 (8.3) N/mm for 3 wt % and 84.7 (7.7) N/mm for 5 wt % aPC3. However, under wet conditions, both the neat composite and aPC3-containing patches showed similar values of 50.7 (6.0), 51.3 (2.1), and 56.6 (2.3) N/mm, respectively. These results are in concordance with the flexural modulus, where mechanical properties of aPC3-containing composites were more affected after the wetting process compared with those of the neat composite. A similar trend was seen with the maximum BS, where under dry conditions, adding aPC3 to the composite formulation increased the strength from 164.4 (12.1) Nmm for the neat composite to 195.1 (28.2) Nmm for the 3 wt % and 295.6 (35.3) Nmm for the 5 wt % aPC3 formulation. Under wet conditions, the BSs of the osteosyntheses made with the three composites were similar. The BS values were normalized with respect to the CSA of the composite patches to account for differences in the dimensions of the patches. After normalization, the differences in BS under dry conditions between the composites were reduced, while under wet conditions, the strengths of the 3 and 5 wt % aPC3 composites, at 9.58 (0.98) and 10.48 (1.32) Nmm/mm^2^, were slightly lower than the neat composite, at 13.67 (1.53) Nmm/mm^2^.

**Table 2:** Mechanical Properties of AdhFix Patches of the Neat Composite and 3 and 5 wt % aPC3 under Dry/Wet Conditions

formulation	conditions	bending stiffness (N/mm)	bending structural stiffness (N/m^2^)	BS (Nmm)	CSA at break (mm^2^)	BS adjusted for CSA (Nmm/mm^2^)
neat	dry	48.0 (5.3)	0.068 (0.008)	164.4 (12.1)	12.57 (0.65)	11.60 (1.03)
aPC3 3 wt %	dry	63.6 (8.3)	0.089 (0.012)	195.1 (28.2)	14.40 (0.84)	13.71 (1.96)
aPC3 5 wt %	dry	84.7 (7.7)	0.119 (0.011)	295.6 (35.3)	19.1 (1.14)	15.41 (1.53)
Neat	wet	50.7 (6.0)	0.071 (0.008)	178.9 (15.5)	13.25 (0.44)	13.67 (1.53)
aPC3 3 wt %	wet	51.3 (2.1)	0.072 (0.003)	165.9 (20.9)	17.19 (0.52)	9.58 (0.98)
aPC3 5 wt %	Wet	56.6 (2.3)	0.080 (0.003)	174.7 (26.1)	16.55 (0.55)	10.48 (1.32)

## 4 Conclusions

Low-molecular-weight isosorbide-based polycarbonates have been successfully synthesized through FPC polymerization and postfunctionalized with allyl functionalities. The presence of these allyl groups allowed for the covalent incorporation of these polymers into pre-existing strong but nondegradable TATO-based composites via TEC chemistry. Raman spectroscopy showed that the polycarbonates did not affect the efficiency of the TEC reaction, as all allyl and thiol groups were consumed after only 10 s of exposure to HEV light. Mechanical testing indicated that the inclusion of aPC1 and aPC2, with terminal allyl groups, did not significantly impact the mechanical properties or hydrolytic degradation of the composite. aPC3, on the other hand, with allyl groups repeated along its backbone, greatly increased the water absorption and degradation of the composite. In the composite containing 5 wt % of aPC3, the water absorption was 290% higher than the prototype composite after 2 weeks in PBS and was 516% higher after 8 weeks. Degradation was improved by 469% after 8 weeks due to the inclusion of aPC3. Adding aPC3 did result in some loss in stiffness, but the 5 wt % aPC3 composite still maintained a modulus above 4 GPa after 8 weeks in PBS and an onset point well above physiological temperature. The polycarbonate-containing composites all outperformed those containing allyl-functionalized PCL (aPCL), the inclusion of which resulted in significantly softer composites but without any improvements to degradation. The 3 and 5 wt % aPC3-containing composites were chosen as the best candidates and used to make osteosyntheses on synthetic bones using the AdhFix approach. The introduction of 3 and 5 wt % aPC3 to the composite improved the stiffness and strength of the osteosyntheses under dry conditions. However, when these patches were tested under wet conditions, the neat composite was not affected, while the aPC3-containing formulations showed a slight decrease in strength when adjusted to the CSA. Although the mechanical properties of the patches with 5 wt % aPC3 decreased after the wetting process, they were similar to the ones for the neat composite under dry conditions. These results, combined with the excellent cytotoxicity profiles, demonstrated that the inclusion of allyl-functionalized polycarbonates is a viable strategy for increasing the degradation of TEC-based composites without sacrificing their mechanical strength and stiffness. The slow degradation and high fixation strength afforded by these polycarbonate-containing composites make them ideal for use in fracture fixation.

## Data Availability

The raw data used to calculate the results in this manuscript are available in the following public repository: doi: 10.5281/zenodo.12689371.

## Supporting Information Available

Synthetic protocols, ^1^H NMR and ^13^C NMR spectra for aPC1, aPC2, and aPCL, characterization, and the formulations used (PDF)
