# Supplementary material for: Enhanced Degradability of Thiol–Ene Composites through the Inclusion of Isosorbide-Based Polycarbonates
Source: ACS Appl Mater Interfaces. 2024 Jul 20;16(30):40056–68. doi: 10.1021/acsami.4c09626 (PMC11299145; doi:10.1021/acsami.4c09626)
Supplement: Supplementary file 1 — am4c09626_si_001.pdf [file am4c09626_si_001.pdf]

Supporting Information

Enhanced degradability of thiol-ene composites through the inclusion of isosorbide-based polycarbonates

Jorge San Jacinto Garcia, Natalia Sanz del Olmo, Daniel J. Hutchinson, Michael Malkoch*

***Corresponding author**: Prof. Michael Malkoch

Contact info: School of Chemistry and Chemical Science, Fibre and Polymer Technology, Coating Technology

Teknikringen 56-58, SE-10044, Stockholm

Fax: (+) 46 (0)8 790 82 83

E-mail: malkoch@kth.se

Table of Contents

SYNTHETIC PROTOCOLS 4

Synthesis of 3- butenoic anhydride: 4

General protocol for polycarbonate synthesis: 5

Scheme S1. Polycarbonate synthetic strategy using step-growth polymerization. 6

Scheme S2. Polycarbonates formation mechanism. 6

Synthesis of allyl-bisMPA co-polycarbonate (aPC3): 6

Polymer post-functionalization: 7

Synthesis of allyl functionalized co-polycarbonate (aPC1): 7

Synthesis of allyl functionalized homo-polycarbonate (aPC2): 8

Synthesis of allyl functionalized polycaprolactone (aPCL): 8

FIGURES 10

Figure S1. ^{1}H-NMR spectrum of aPC1 in $CDCl_3$. 10

Figure S2. ^{13}C-NMR spectrum of aPC1 in $CDCl_3$. 10

Figure S3. ^{1}H-NMR spectrum of aPC2 in $CDCl_3$. 11

Figure S4. ^{13}C-NMR spectrum of aPC2 in $CDCl_3$. 11

Figure S5. 1H-NMR spectrum of aPC3 in CDCl3 12

Figure S6. ^{13}C-NMR spectrum of aPC3 in $CDCl_3$. 12

Figure S7. ^{1}H-NMR spectrum of aPCL in $CDCl_3$. 13

Figure S8. ^{13}C-NMR spectrum of aPCL in $CDCl_3$. 14

Figure S9. Scanning Electron Microscopy (SEM) images of the composite cross-section with a magnification of 50x. 14

TABLES. 14

Table S1. Molecular weight (M_n), polydispersity (PDI) and T_g of the different allyl functionalized polymers. 14

Table S2. Mass of components used for the different formulations evaluated. 15

Table S3. Contact angle of the different formulation at time 0 weeks and 8 weeks. 15

Table S4. Properties of all formulations evaluated. 16

SYNTHETIC PROTOCOLS

Synthesis of 3- butenoic anhydride:

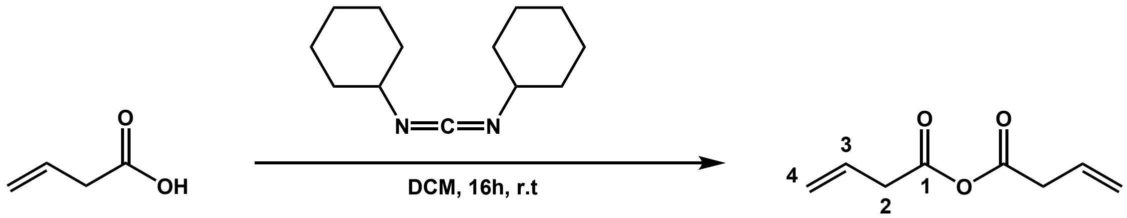

Butenoic acid (3.00 g, 34.8 mmol) was dissolved in DCM (10 mL) in a round bottom flask. Then, DCC (3.60 g, 17.4 mmol) was dissolved in DCM (10 mL) and added dropwise to the butenoic acid solution while stirring. The reaction mixture was left to stir overnight at room temperature and the following day, after verifying through NMR the conversion into anhydride, the reaction mixture was filtered through celite, removing the excess of DCU formed as byproduct. The resultant solution was then evaporated and redissolved in a mixture 50/50 of EtOAc/heptane. This mixture was heated until ebullition and then cooled down in a fridge overnight. After that, the solution was filtered through celite and evaporated in the rotavap and then under vacuum overnight. 3- butenoic anhydride was afforded as a colorless oil (4.59 g, 85%). ^{1}H NMR (400 MHz, $CDCl_3$) δ 5.89 (2H, ddt, *J = 17, 10, 7 Hz*, H3), 5.30 – 5.10 (4H, m, H4), 3.24 (4H, dt, *J = 7, 1 Hz*, H2). ^{13}C NMR (101 MHz, CDCl3) δ 167.07 (C1), 128.34 (C3), 120.22 (C4), 39.84 (C2).

General protocol for polycarbonate synthesis:

A round bottom flask (100 mL) with a stir bar was charged with CsF (64 mg, 4.27 mmol) and heated under vacuum. Then, the diol, either neopentyl glycol (0.89 g, 8.55 mmol; PC1), isosorbide (1.25 g, 8.55 mmol; PC2) or allyl-bisMPA (1.49 g, 8.55 mmol; aPC3), and bis-carbonylimidazole isosorbide (3.00g, 8.97 mmol) were added and subjected to three vacuum/Argon cycles. Lastly, solvent (9 mL) was added and the reaction mixture was left to stir overnight (16h) with a temperature dependent of the solvent used (rt for $CHCl_3$ and DCM, 45 ^{o}C for THF and 60 ^{o}C for DMF). After 16 h, a crude aliquot was taken and analyzed to measure monomer conversion through ^{1}H-NMR ($CDCl_3$), molecular number weight (Mn) and PDI through DMF-SEC. The polymer was purified through the precipitation of the crude mixture into stirring Et_2O (for $CHCl_3$ and DCM) or distilled water (for THF and DMF) (500 mL). The precipitated polymer was then filtered, washed and dried in a vacuum oven at 50 □ C.

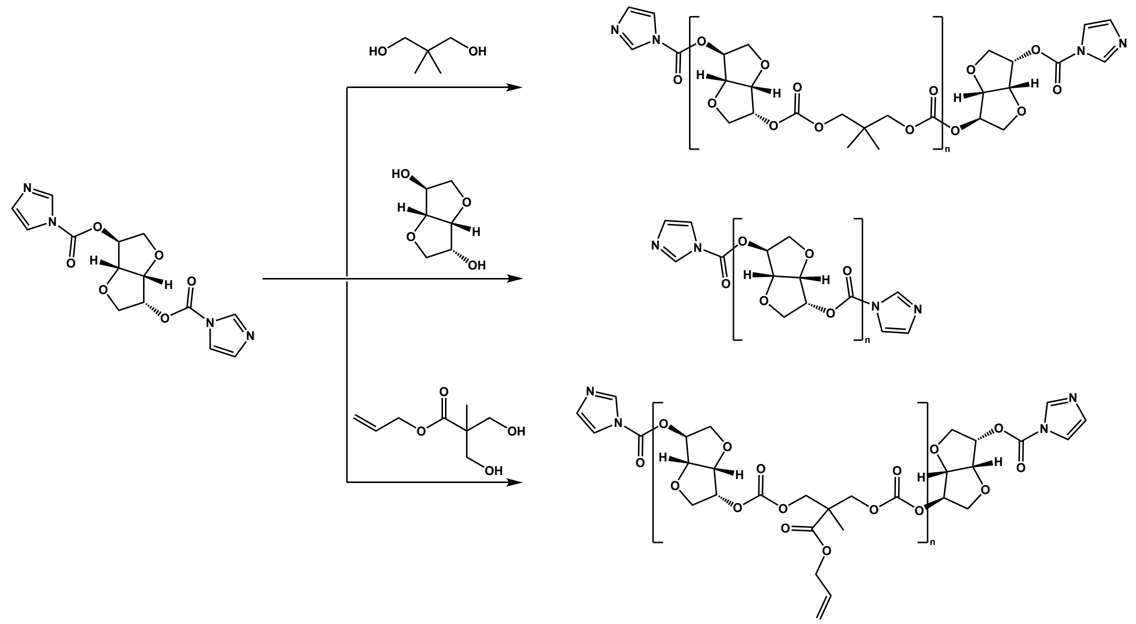

Scheme S1. Polycarbonate synthetic strategy using step-growth polymerization.

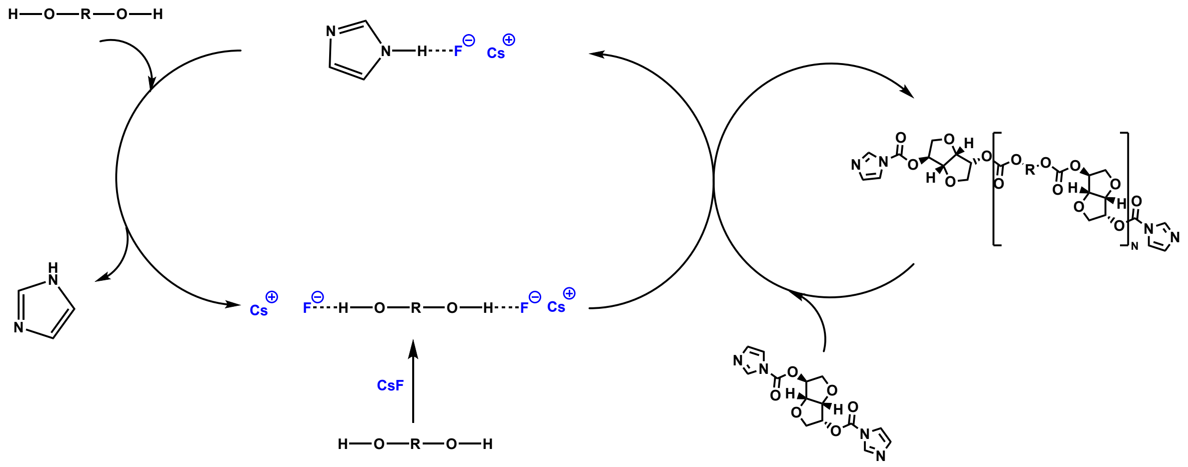

Scheme S2. Polycarbonates formation mechanism.

Synthesis of allyl-bisMPA co-polycarbonate (aPC3):

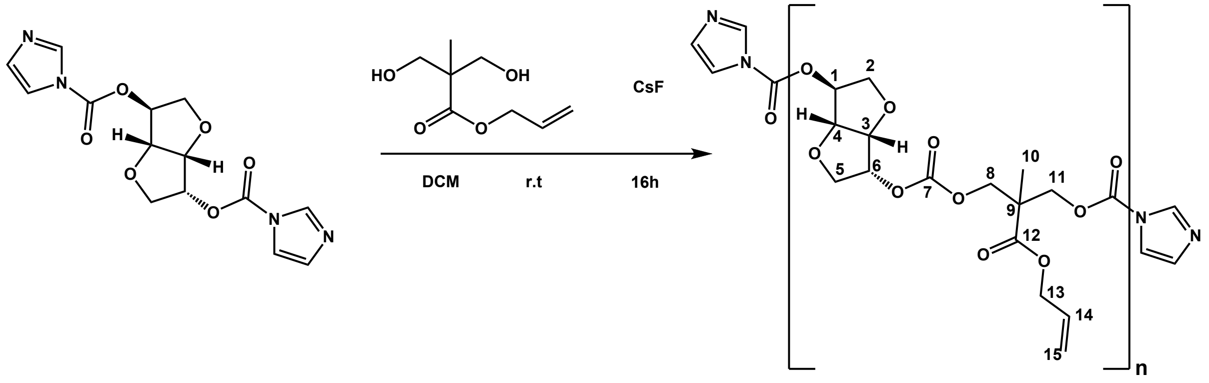

aPC3 was synthesized following the general protocol described above using DCM as solvent. The polymer was afforded as a white powder after precipitation in Et_2O and dried overnight in a vacuum oven at 50 °C (3.2 g, 71%). ^1H NMR (400 MHz, $CDCl_3$) δ ppm: 5.87 (1H, ddt, *J = 17.5, 9.7, 5.9, Hz*, H14), 5.38 – 5.19 (2H, m, H15), 5.08 (2H, dt, *J = 13.4, 4.0 Hz*, H1, H6), 4.92 – 4.81 (1H, m, H4), 4.61 (2H, d, *J = 5.6 Hz*, H13), 4.57 – 4.47 (1H, m, H3), 4.43 – 4.18 (4H, m, H8, H11),

4.14 – 3.81 (4H, m, H2, H5), 1.30 – 1.17 (3H, m, H10). ^{13}C-NMR (101 MHz, $CDCl_3$) δ ppm: 171.8 (C12), 154.4, 153.6 (C7), 131.5 (C14), 118.6 (C15), 85.7 (C4), 81.5 (C1), 80.9 (C3), 73.0 (C2), 70.6 (C5), 68.6 (C13), 65.9 (C8, C11), 46.5 (C9), 17.5 (C10). M_n SEC (DMF): 4.2 kDa, PDI= 2.2, DSC: T_g = 93 °C.

Polymer post-functionalization:

Synthesis of allyl functionalized co-polycarbonate (aPC1):

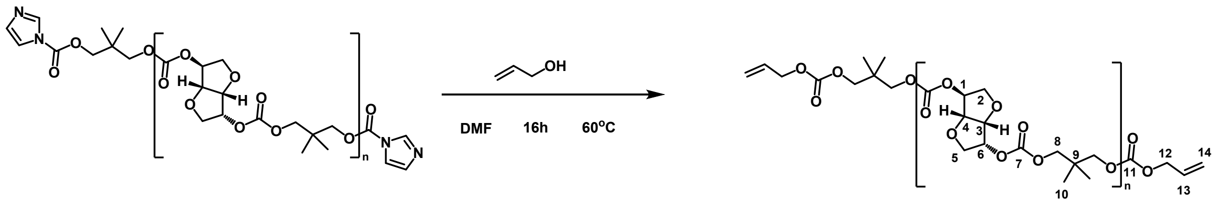

In a round bottom flask (100 mL) with a stir bar the polycarbonate (5.36 g, 0.568 mmol) was dissolved in DMF (15 mL). The mixture was heated at 60°C and then allyl alcohol (0.066 g, 1.1 mmol) was added dropwise. The reaction mixture was left to stir overnight and then precipitated in distilled water (500 mL), filtered and dried in a vacuum oven at 50 °C. Allyl functionalized polycarbonate was afforded as white powder (4.82 g, 90%). ^{1}H NMR (400 MHz, $CDCl_3$) δ ppm: 6.01 – 5.82 (2H, m, H13), 5.41 – 5.21 (4H, m, H14), 5.08 (48H, dt, *J = 9.0, 4.0 Hz*, H1, H6), 4.92 – 4.83 (24H, m, H3), 4.52 (24H, dd, *J = 15.7, 5.1 Hz*, H4), 4.17 – 3.77 (128H, m, H2, H5, H8), 0.98 (42H, d, *J = 2.4 Hz*, H10). ^{13}C NMR (101 MHz, $CDCl_3$) δ ppm: 153.9, 153.6, 153.2 (C7), 85.7, 85.7 (C4), 81.5 (C1), 80.9 (C3), 77.1 (C6), 73.1 (C2), 73.0 (C5), 70.6, 70.6 (8), 35.1, 35.1 (C9), 21.4 (C10). M_n SEC (DMF) = 3.79 kDa; PDI= 1.92. DSC: T_g= 112 °C.

Synthesis of allyl functionalized homo-polycarbonate (aPC2):

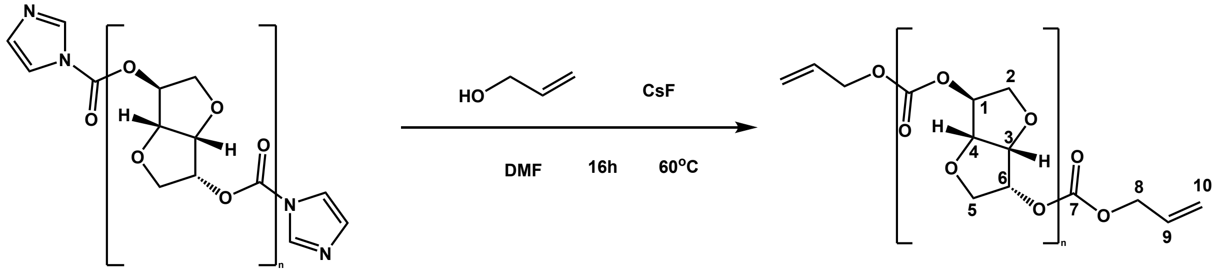

In a round bottom flask (100 mL) with a stir bar the polycarbonate (9.08 g, 0.934 mmol) was dissolved in DMF (25 mL). The mixture was heated at 60°C and then allyl alcohol (0.11 g, 1.9 mmol) was added dropwise. The reaction mixture was left to stir overnight and then precipitated in distilled water (1 L), filtered and dried in a vacuum oven at 50°C. Allyl functionalized polycarbonate was afforded as white powder (7.85 g, 87%). ^{1}H NMR (400 MHz, $CDCl_3$) δ ppm: 5.90 (2H, tdd, *J = 16.5, 7.8, 4.0 Hz*, H9), 5.42 – 5.23 (4H, m, H10), 5.14 – 4.99 (52H, m, H1, H6), 4.90 – 4.81 (26H, m, H4), 4.51 (26H, dd, *J = 15.3, 4.9 Hz*, H3), 4.40 – 4.24 (4H, m, H8), 4.10 – 3.80 (104H, m, H2, H5). ^{13}C NMR (101MHz, $CDCl_3$) δ ppm: 153.9, 153.6, 153.2 (C7), 85.8 (C4), 81.5, 81.4 (C1), 81.0, 80.9 (C3), 77.2, 77.1 (C6), 73.1, 73.0 (C2), 70.6, 70.5, 70.5 (C5). M_n SEC (DMF) = 2.91 kDa; PDI= 1.64. DSC: T_g= 152 °C.

Synthesis of allyl functionalized polycaprolactone (aPCL):

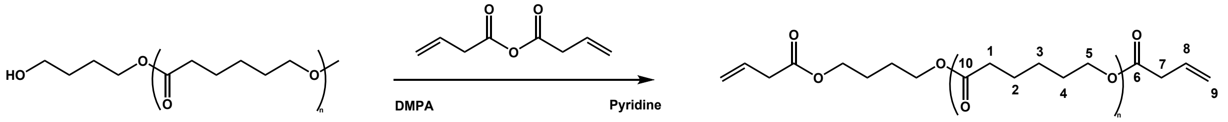

Polycaprolactone (20.00g, 5.00 mmol) was added together with pyridine (25 mL) in a round bottom flask. Once the polymer was completely dissolved, DMPA (0.77 g, 2 mmol) and 3-butenoic anhydride (3.08 g, 20 mmol) were added to the mixture. The reaction was left to stir

overnight and then it was precipitated in MeOH (1 L). The desired polymer was afforded as a slightly brownish powder that then was dried in a vacuum oven at 50 °C (19.2 g, 93%). ^{1}H NMR (400 MHz, $CDCl_3$) δ ppm: 5.85 (2H, ddt, *J = 17, 10, 7 Hz*, H8), 5.10 (4H, m, H9), 3.99 (101H t, *J = 7 Hz*, H5), 3.02 (4H, dt, *J = 7, 2 Hz*, H7), 2.24 (98H, t, *J = 7 Hz*, H1), 1.58 (197H, dqd, *J = 10, 7, 3 Hz*, H2, H4), 1.32 (97H, m, H3). ^{13}C NMR (101 MHz, $CDCl_3$) δ ppm: 173.48 (C10), 171.51 (C6), 130.34 (C8), 118.44 (C9), 64.09 (C5), 39.12 (C7), 34.07 (C2), 28.31 (C4), 25.49 (C3), 24.54 (C2). SEC: M_n= 4.89 kDa; D= 1.22. DSC: T_g= 52 °C.

FIGURES

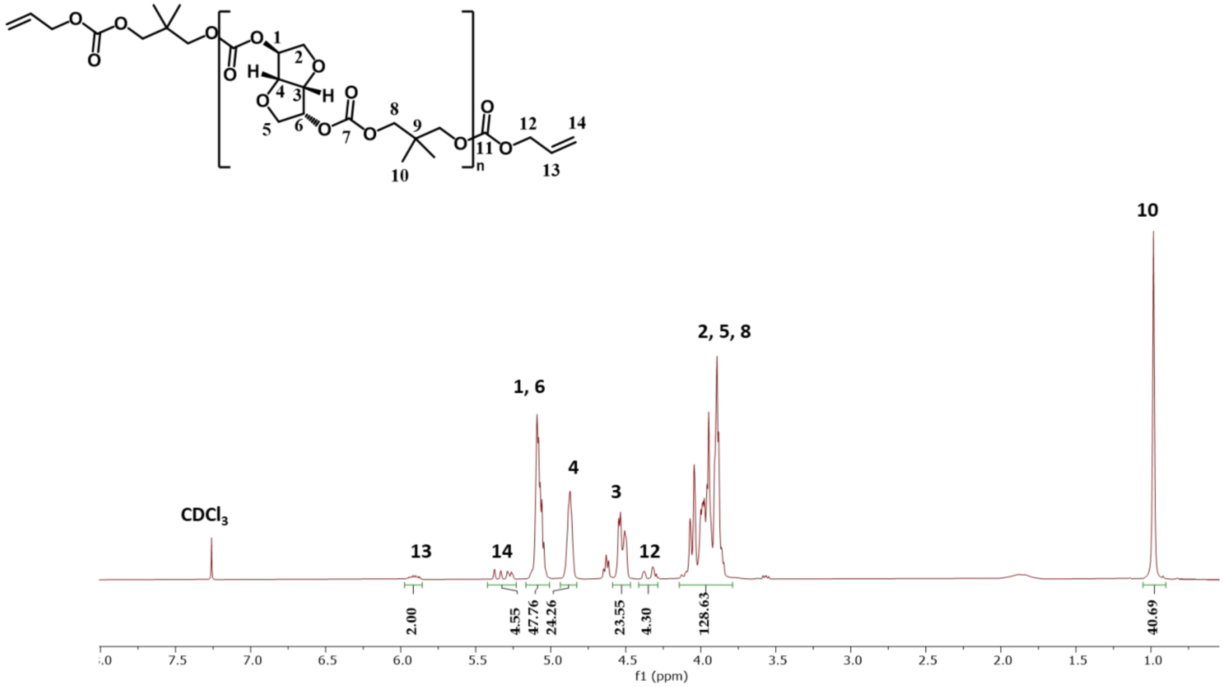

Figure S1. ^{1}H-NMR spectrum of aPC1 in $CDCl_3$.

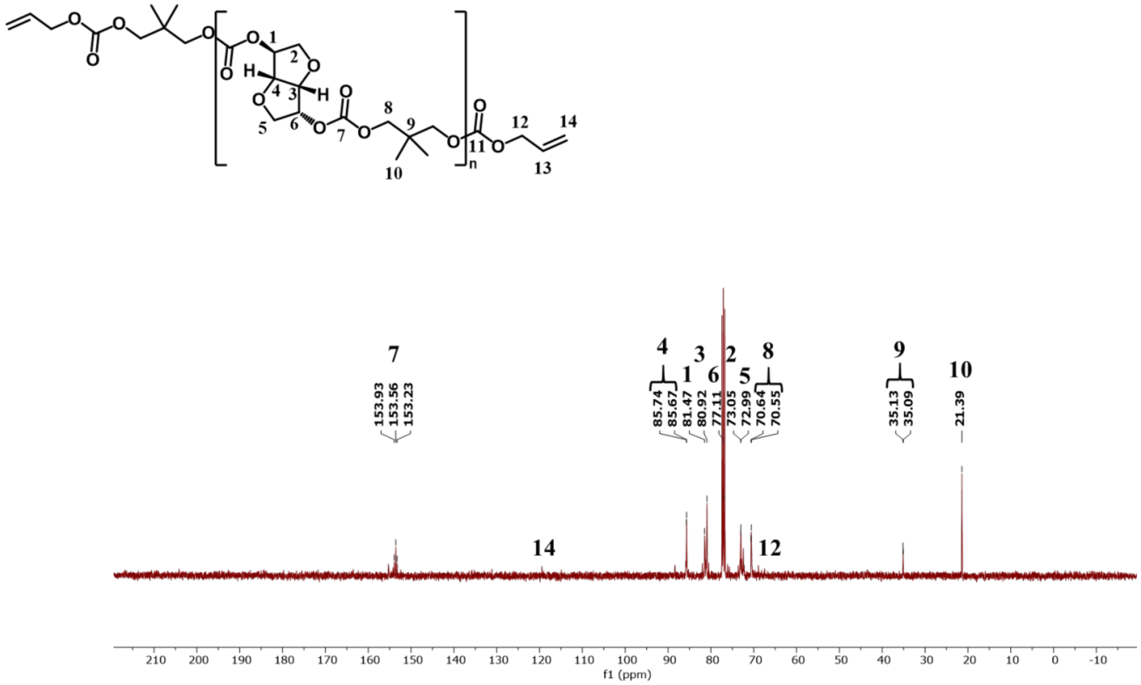

Figure S2. ^{13}C-NMR spectrum of aPC1 in $CDCl_3$.

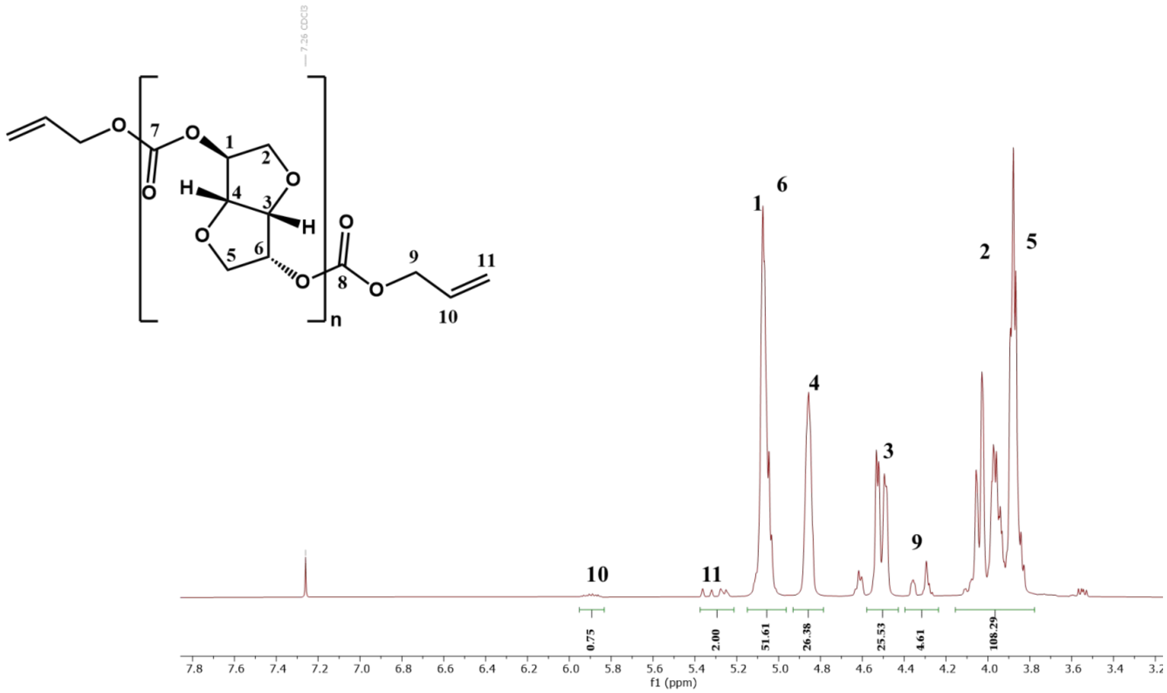

Figure S3. ^{1}H-NMR spectrum of aPC2 in $CDCl_3$.

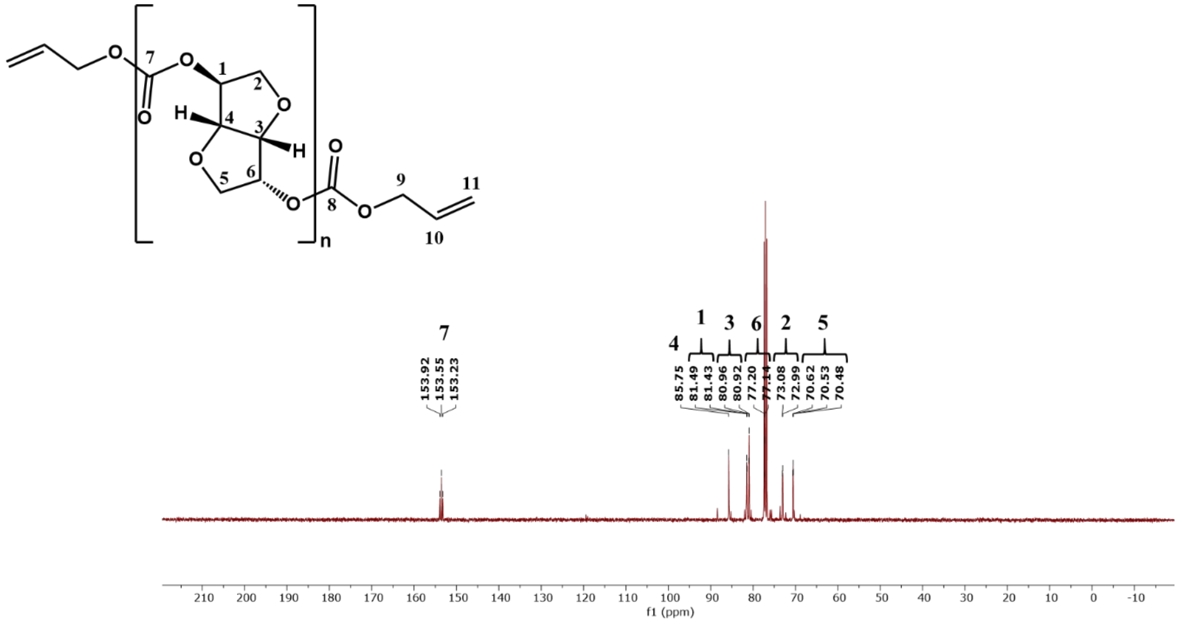

Figure S4. ^{13}C-NMR spectrum of aPC2 in $CDCl_3$.

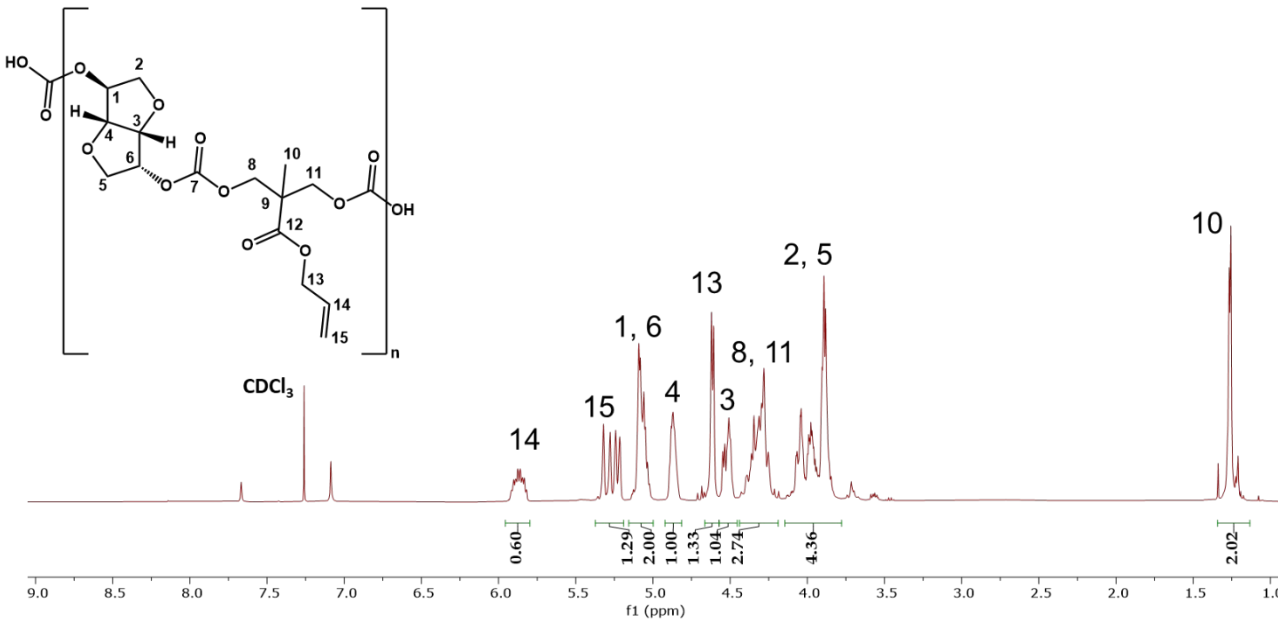

Figure S5. 1H-NMR spectrum of aPC3 in CDCl3.

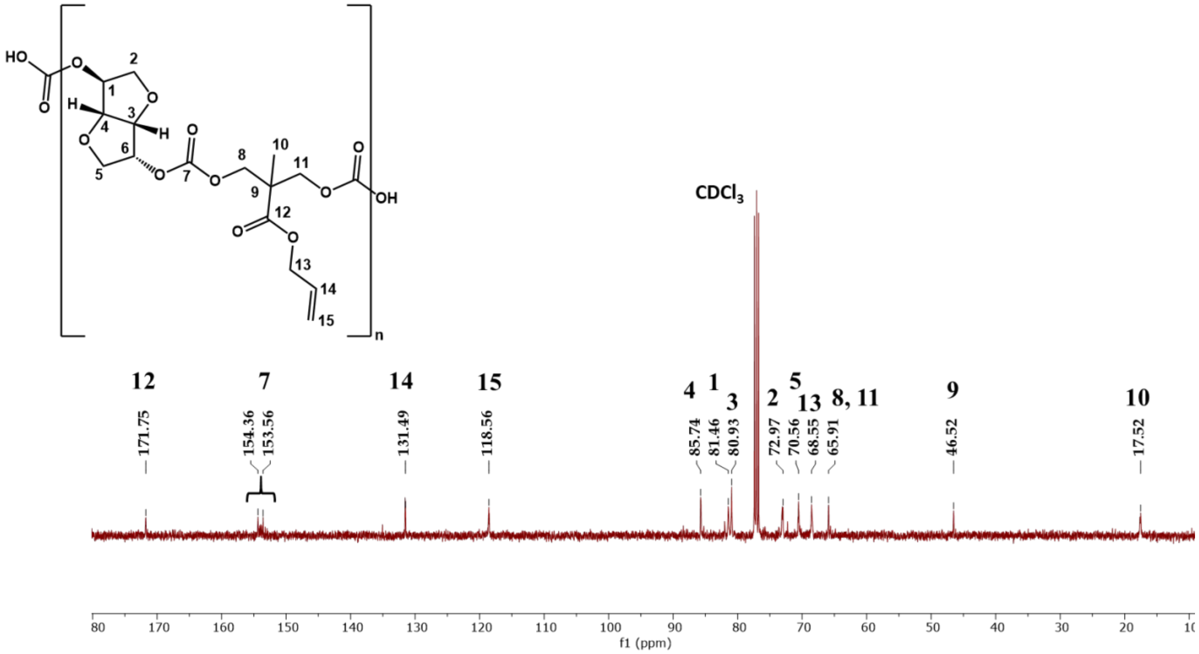

Figure S6. ^{13}C-NMR spectrum of aPC3 in $CDCl_3$.

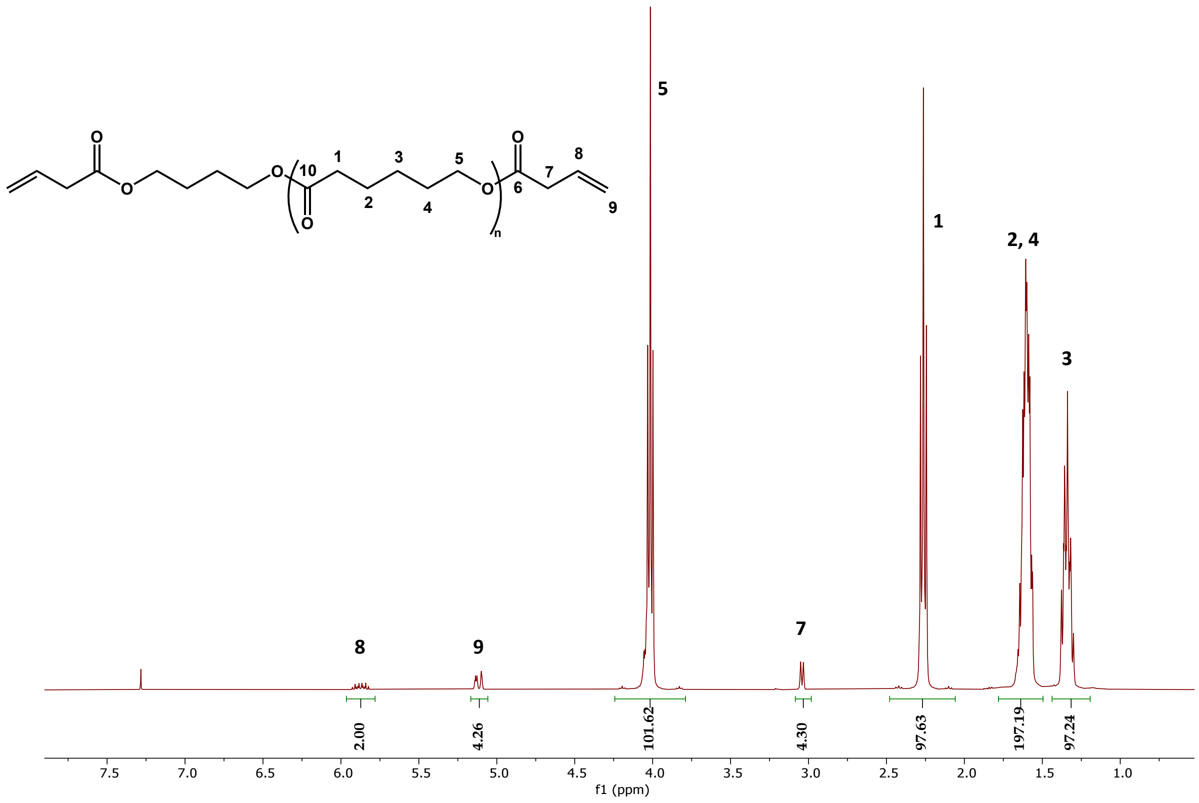

Figure S7. ¹H-NMR spectrum of aPCL in $CDCl_3$.

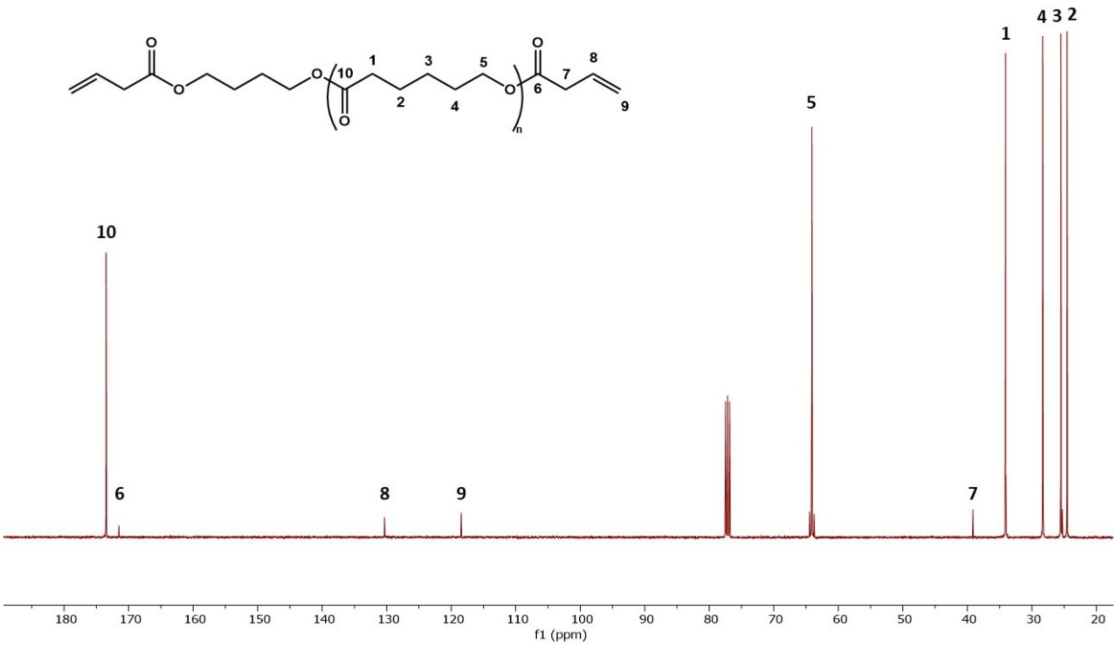

Figure S8. ^{13}C-NMR spectrum of aPCL in $CDCl_3$.

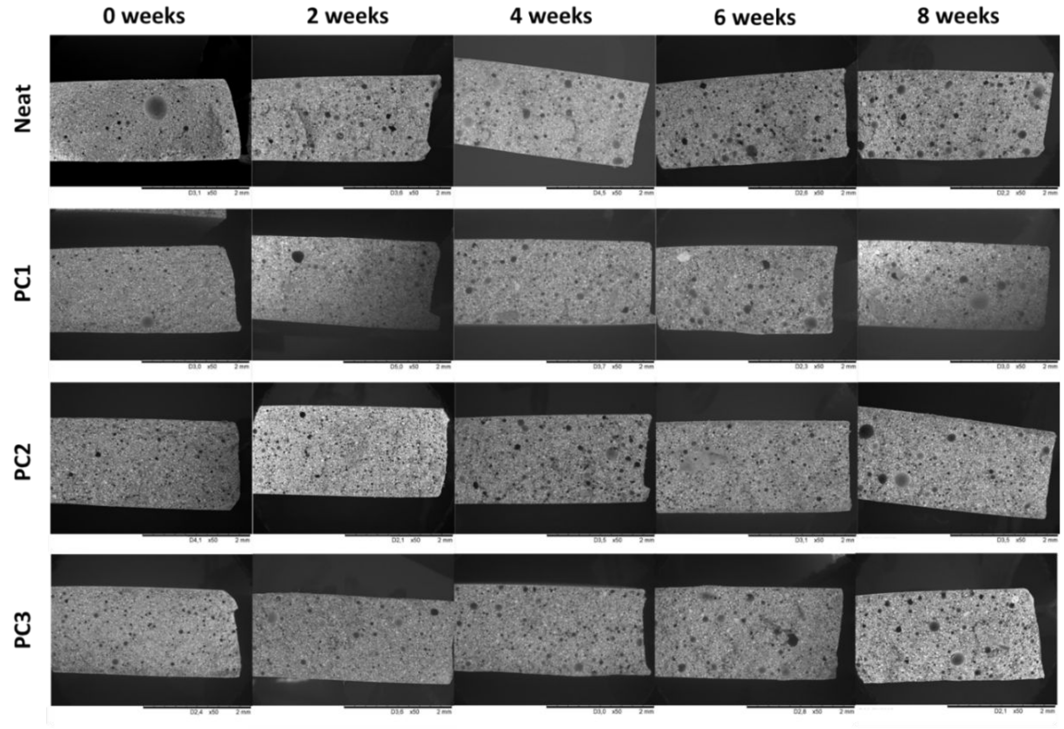

Figure S9. Scanning Electron Microscopy (SEM) images of the composite cross-section with a magnification of 50x.

TABLES.

Table S1. Molecular weight (M_n), polydispersity (PDI) and T_g of the different allyl functionalized polymers.

Polymer	Mn (kDa)	PDI	T_g (°C)
aPC1	3.8	1.9	112
aPC2	2.9	1.6	152
aPC3	4.2	2.2	91
aPCL	4.1	2.3	52

Table S2. Mass of components used for the different formulations evaluated.

	Polymer	wt %	TMTATO (mg)	TATATO (mg)	Degradable Polymer (mg)	Catalyst 57 (mg)	TPO (mg)	HA (mg)
Neat	None	0	1086	746.9	0	42	10.68	2400
PC1		1	1086	745	45	42	10.68	2400
		3	1086	741	134	42	10.68	2400
		5	1086	737	225	42	10.68	2400
PC2		1	1086	744.5	44	42	10.68	2400
		3	1086	740	133	42	10.68	2400
		5	1086	734.5	225	42	10.68	2400
PC3		1	1086	742	45	42	10.68	2400
		3	1086	730	134	42	10.68	2400
		5	1086	718	225	42	10.68	2400
Allyl PCL		1	1086	745	45	42	10.68	2400
		3	1086	742	134	42	10.68	2400
		5	1086	738	225	42	10.68	2400

Table S3. Contact angle of the different formulation at time 0 weeks and 8 weeks.

		Neat composite	PC1	PC2	PC3	PCL
Time	0 weeks	58.9 (2.5)	51.1 (2.3)	58.5 (2.4)	49.8 (1.7)	72.8 (0.8)
	8 weeks	52.4 (0.2)	52.0 (1.1)	55.4 (0.8)	49.1 (2.9)	72.2 (0.5)

Table S4. Properties of all formulations evaluated.

	Polymer	wt %	Time	E_f (MPa)	σ_f (MPa)	T_g (°C)	Onset point (°C)	Water Absorption (%)	Degradation (%)	Contact Angle (degrees)	Porosity (%)
Neat	None	0	0	6824 (66)	64 (1)	84 (1)	58 (1)	-	-	59 (3)	2.54 (0.27)
			2	5697 (84)	43 (1)	82 (1)	61 (1)	1.36 (0.01)	0.01 (0.01)	-	3.37 (0.21)
			4	5449 (89)	43 (1)	83 (1)	61 (1)	1.43 (0.01)	0.21 (0.01)	-	3.90 (0.42)
			6	5371 (68)	44 (1)	82 (1)	63 (1)	1.54 (0.02)	0.29 (0.01)	-	3.23(0.32)
			8	5551 (37)	45 (1)	90 (1)	65 (2)	1.61 (0.02)	0.31 (0.01)	52 (1)	4.30 (0.20)
PC1		1	0	7206 (134)	68 (2)	-	-	-	-	-	-
			2	5834 (82)	56 (1)	-	-	1.26 (0.02)	0.18 (0.03)	-	-
			4	5313 (70)	39 (1)	-	-	1.46 (0.03)	0.31 (0.02)	-	-
			6	5532 (38)	43 (1)	-	-	1.50 (0.03)	0.41 (0.02)	-	-
			8	5426 (70)	42 (1)	-	-	1.60 (0.04)	0.45 (0.03)	-	-
		3	0	6469 (150)	65 (2)	-	-	-	-	-	-
			2	5873 (54)	52 (1)	-	-	1.37 (0.01)	0.16 (0.02)	-	-
			4	5494 (43)	41 (1)	-	-	1.47 (0.01)	0.40 (0.02)	-	-
			6	5258 (43)	40 (1)	-	-	1.59 (0.01)	0.44 (0.01)	-	-
			8	5242 (69)	40 (1)	-	-	1.63 (0.01)	0.43 (0.01)	-	-
		5	0	6660 (192)	67 (2)	82 (1)	55 (1)	-	-	51 (2)	1.83 (0.18)
			2	5648 (53)	53 (1)	82 (1)	62 (1)	1.45 (0.01)	0.17 (0.01)	-	2.65 (0.31)
			4	5296 (30)	40 (1)	84 (1)	63 (1)	1.50 (0.03)	0.32 (0.01)	-	3.60 (0.21)
			6	5246 (49)	39 (1)	81 (1)	62 (1)	1.65 (0.01)	0.45 (0.01)	-	3.31 (0.20)
			8	5293 (115)	39 (2)	81 (1)	62 (1)	1.72 (0.01)	0.43 (0.01)	52 (1)	5.18 (0.54)
PC2		1	0	7103 (90)	65 (3)	-	-	-	-	-	-
			2	5523 (75)	50 (1)	-	-	1.40 (0.06)	0.19 (0.03)	-	-
			4	5298 (118)	44 (2)	-	-	1.57 (0.04)	0.31 (0.01)	-	-
			6	5269 (95)	44 (1)	-	-	1.57 (0.02)	0.44 (0.01)	-	-
			8	5509 (41)	46 (1)	-	-	1.65 (0.02)	0.45 (0.01)	-	-
		3	0	6929 (106)	70 (2)	-	-	-	-	-	-
			2	5646 (44)	51 (1)	-	-	1.34 (0.01)	0.23 (0.03)	-	-
			4	5295 (89)	42 (1)	-	-	1.59 (0.01)	0.44 (0.01)	-	-
			6	5438 (94)	46 (1)	-	-	1.57 (0.02)	0.58 (0.01)	-	-
			8	5338 (101)	44 (2)	-	-	1.68 (0.02)	0.55 (0.01)	-	-
		5	0	6471 (71)	71 (2)	82 (1)	51 (1)	-	-	59 (2)	3.82 (0.25)
			2	5701 (53)	52 (1)	83 (1)	61 (1)	1.38 (0.05)	0.21 (0.02)	-	3.58 (0.23)
			4	4766 (105)	40 (1)	81 (1)	61 (1)	1.59 (0.03)	0.39 (0.01)	-	4.39 (0.20)
			6	5417 (64)	46 (2)	84 (1)	63 (1)	1.57 (0.04)	0.53 (0.01)	-	4.62 (0.37)

		8	4672 (117)	40 (1)	84 (1)	64 (1)	1.68 (0.04)	0.55 (0.02)	55 (1)	6.03 (0.30)
PC3	1	0	7039 (94)	69 (1)	-	-	-	-	-	-
		2	5601 (51)	52 (1)	-	-	1.69 (0.03)	0.27 (0.03)	-	-
		4	5163 (108)	45 (1)	-	-	1.89 (0.02)	0.47 (0.02)	-	-
		6	5070 (61)	44 (1)	-	-	2.13 (0.02)	0.51 (0.01)	-	-
		8	5355 (47)	49 (2)	-	-	2.85 (0.07)	0.53 (0.05)	-	-
	3	0	6952 (93)	62 (2)	-	-	-	-	-	-
		2	4506 (24)	43 (1)	-	-	2.59 (0.04)	0.59 (0.02)	-	-
		4	4526 (59)	43 (1)	-	-	3.05 (0.05)	0.85 (0.03)	-	-
		6	4362 (22)	41 (1)	-	-	3.42 (0.07)	0.91 (0.01)	-	-
		8	4537 (36)	43 (1)	-	-	5.18 (0.29)	0.91 (0.05)	-	-
	5	0	6524 (88)	65 (2)	85 (1)	55 (1)	-	-	50 (2)	3.16 (0.28)
		2	4047 (39)	46 (1)	86 81)	62 (1)	3.94 (0.06)	1.02 (0.02)	-	3.03 (0.20)
		4	4019 (157)	40 (1)	85 (1)	61 (1)	4.59 (0.10)	1.43 (0.04)	-	3.70 (0.13)
		6	4172 (47)	43 (1)	87 (1)	66 (1)	5.33 (0.20)	1.55 (0.06)	-	3.96 (0.29)
		8	4016 (28)	41 (1)	89 (1)	63 (1)	8.31 (0.18)	1.60 (0.02)	49 (3)	6.94 (0.51)
PCL	1	0	6090 (62)	62 (2)	-	-	-	-	-	-
		2	4501 (45)	45 (1)	-	-	1.38 (0.01)	0.37 (0.01)	-	-
		4	4537 (101)	45 (1)	-	-	1.48 (0.02)	0.31 (0.01)	-	-
		6	4794 (79)	47 (1)	-	-	1.57 (0.03)	0.29 (0.01)	-	-
		8	4914 (88)	47 (1)	-	-	1.62 (0.07)	0.34 (0.02)	-	-
	3	0	5682 (61)	61 (1)	-	-	-	-	-	-
		2	3335 (44)	44 (1)	-	-	1.38 (0.01)	0.26 (0.01)	-	-
		4	3242 (62)	49 (1)	-	-	1.47 (0.01)	0.25 (0.01)	-	-
		6	3534 (80)	43 (1)	-	-	1.5 (0.02)	0.22 (0.02)	-	-
		8	3794 (128)	45 (1)	-	-	1.55 (0.02)	0.35 (0.02)	-	-
	5	0	4469 (56)	56 (2)	69 (1)	42 (2)	-	-	73 (1)	-
		2	1996 (39)	39 (1)	71 (1)	44 (1)	1.27 (0.03)	0.21 (0.01)	-	-
		4	2026 (37)	41 (1)	69 (1)	43 (1)	1.39 (0.03)	0.22 (0.02)	-	-
		6	2247 (64)	42 (1)	69 (1)	42 (1)	1.41 (0.03)	0.28 (0.01)	-	-
		8	2271 (56)	41 (1)	64 (1)	35 (1)	1.42 (0.02)	0.27 (0.01)	72 (1)	-

Data repository.

The raw data used to calculate the results in this manuscript are available in the following public repository: doi: 10.5281/zenodo.12689371.
